# Structure-based design of a single-chain triple-disulfide-stabilized fusion-glycoprotein trimer that elicits high-titer neutralizing responses against human metapneumovirus

**DOI:** 10.1371/journal.ppat.1011584

**Published:** 2023-09-22

**Authors:** Li Ou, Steven J. Chen, I-Ting Teng, Lijuan Yang, Baoshan Zhang, Tongqing Zhou, Andrea Biju, Cheng Cheng, Wing-Pui Kong, Nicholas C. Morano, Erik-Stephane D. Stancofski, John-Paul Todd, Yaroslav Tsybovsky, Shuishu Wang, Cheng-Yan Zheng, John R. Mascola, Lawrence Shapiro, Ruth A. Woodward, Ursula J. Buchholz, Peter D. Kwong

**Affiliations:** 1 Vaccine Research Center, National Institutes of Health, Bethesda, Maryland, United States of America; 2 Laboratory of Infectious Diseases, National Institute of Allergy and Infectious Diseases, Bethesda, Maryland, United States of America; 3 Zuckerman Mind Brain Behavior Institute, Columbia University, New York, New York, United States of America; 4 Department of Biochemistry and Molecular Biophysics, Columbia University Vagelos College of Physicians and Surgeons, New York, New York, United States of America; 5 Electron Microscopy Laboratory, Cancer Research Technology Program, Frederick National Laboratory for Cancer Research, Frederick, Maryland, United States of America; Thomas Jefferson University - Center City Campus: Thomas Jefferson University, UNITED STATES

## Abstract

The Pneumoviridae family of viruses includes human metapneumovirus (HMPV) and respiratory syncytial virus (RSV). The closely related Paramyxoviridae family includes parainfluenza viruses (PIVs). These three viral pathogens cause acute respiratory tract infections with substantial disease burden in the young, the elderly, and the immune-compromised. While promising subunit vaccines are being developed with prefusion-stabilized forms of the fusion glycoproteins (Fs) of RSV and PIVs, for which neutralizing titers elicited by the prefusion (pre-F) conformation of F are much higher than for the postfusion (post-F) conformation, with HMPV, pre-F and post-F immunogens described thus far elicit similar neutralizing responses, and it has been unclear which conformation, pre-F or post-F, would be the most effective HMPV F-vaccine immunogen. Here, we investigate the impact of further stabilizing HMPV F in the pre-F state. We replaced the furin-cleavage site with a flexible linker, creating a single chain F that yielded increased amounts of pre-F stabilized trimers, enabling the generation and assessment of F trimers stabilized by multiple disulfide bonds. Introduced prolines could increase both expression yields and antigenic recognition by the pre-F specific antibody, MPE8. The cryo-EM structure of a triple disulfide-stabilized pre-F trimer with the variable region of antibody MPE8 at 3.25-Å resolution confirmed the formation of designed disulfides and provided structural details on the MPE8 interface. Immunogenicity assessments in naïve mice showed the triple disulfide-stabilized pre-F trimer could elicit high titer neutralization, >10-fold higher than elicited by post-F. Immunogenicity assessments in pre-exposed rhesus macaques showed the triple disulfide-stabilized pre-F could recall high neutralizing titers after a single immunization, with little discrimination in the recall response between pre-F and post-F immunogens. However, the triple disulfide-stabilized pre-F adsorbed HMPV-directed responses from commercially available pooled human immunoglobulin more fully than post-F. Collectively, these results suggest single-chain triple disulfide-stabilized pre-F trimers to be promising HMPV-vaccine antigens.

## Author summary

Immune responses to the fusion (F) glycoprotein trimer of human metapneumovirus (HMPV) could form the basis of an effective vaccine. However, the F glycoprotein assumes two conformations or shapes, prefusion (pre-F) and postfusion (post-F), and the shape of the most effective HMPV-vaccine immunogen has been unclear. Here, we use structure-based design to create a single chain version of F, which we stabilized in the pre-F shape with three additional disulfide bonds and assessed structurally, antigenically, and immunogenically. Structurally, we confirmed by cryo-electron microscopy that the three designed disulfides formed. Antigenically, the single-chain triple disulfide-stabilized F trimer was well recognized by the pre-F specific antibody, MPE8. And immunogenically, the single-chain triple disulfide-stabilized F trimer elicited high titer neutralizing responses, both in naïve mice and in pre-exposed rhesus macaques. Overall, our results suggest a highly stabilized pre-F trimer, such as the single-chain triple disulfide-stabilized F trimer that we described here, may be a highly effective HMPV-vaccine immunogen.

## Introduction

The *Pneumoviridae* family of viruses includes respiratory syncytial virus (RSV) and human metapenumovirus (HMPV). The *Paramyxoviridae* family includes several important human pathogens, such as mumps virus, measles virus, and parainfluenza viruses (PIVs) [1–9]. While effective vaccines against mumps and measles have been licensed and widely used [10], and recently two RSV vaccines have been approved by US Food & Drug Administration (FDA), there is currently no licensed vaccine for HMPV or PIVs. These viral pathogens infect nearly everyone during childhood, induce acute respiratory tract infections, and are leading causes of hospitalization in the United States for infants [11], with substantial disease burdens in the elderly and the immune compromised [12].

One promising vaccine target against RSV, HMPV, and PIV is their fusion (F) glycoprotein trimer, which is the target of potent virus-neutralizing antibodies. Merger of viral membrane and target host membrane is the critical functional step performed by the F glycoprotein, a type 1 fusion machine [13], to enable entry of the enveloped virion. This merger involves the F glycoprotein switching conformations, from prefusion (pre-F), through a pre-hairpin intermediate to the postfusion (post-F) state [14]. With RSV and PIVs types 1–4, pre-F elicits much higher neutralizing responses than post-F [15–19]. However, with HMPV, 1^st^-generation pre-F and post-F immunogens elicit similar neutralizing responses [20,21] as did 2^nd^-generation interprotomer-disulfides stabilized versions [22].

Here we further stabilize the HMPV trimer in pre-F conformation and assess these stabilized variants for their antigenicity and immunogenicity. We were able to improve the yield of stabilized HMPV F by linking the two subunits of F as a single chain. Additionally, we conducted screenings of F variants, which were stabilized in the pre-F conformation through the incorporation of multiple disulfides and prolines. The structure of one of the best variants with triple-disulfide stabilization was then determined in complex with the pre-F specific antibody MPE8 [23]. We observed this triple-stabilized variant to elicit especially high HMPV-neutralizing responses in both naïve mice and pre-exposed rhesus macaques and, furthermore, showed this triple-stabilized variant could more fully adsorb F recognition from human Flebogamma. Overall, our results suggest that a single-chain triple-disulfide-stabilized F trimer may be a highly effective HMPV-vaccine immunogen.

## Materials and methods

### Ethics statement

Mouse experiments and nonhuman primate (NHP) animal studies were reviewed and approved by the Animal Care and Use Committee of the Vaccine Research Center, National Institutes of Allergy and Infectious Diseases (NIAID), National Institutes of Health (NIH). Animals were housed and cared for in accordance with local, state, federal and institute policies within an Association for Assessment and Accreditation of Laboratory Animal Care (AAALAC) International accredited facility at the NIH.

### Structure-based design of pre-F HMPV F trimer

Prefusion HMPV F variants were derived from the HMPV F v3B [22], which comprises HMPV F residues 1–485 with a C-terminal T4 fibritin trimerization motif (foldon), thrombin site, His6-tag, and Strep-tag. We used the structure of pre-F HMPV F (PDB: 5WB0) [21] to design single-chain, disulfide-stabilized, and proline-stabilized variants of HMPV F. We designed 9 F2-F1 linker variants, 17 disulfide bonds, 18 helix-disrupting proline mutations or proline combinations, 21 combinations of disulfides and cavity-filling mutations with optimal F2-F1 linker (S1 Table). We used three antibodies to evaluate the antigenicity. MPE8, known as a site III-targeting antibody, only binds to prefusion conformation. Two additional antibodies, MPE33 (site IV-targeting) and MPF5 (binding site undefined), can bind to both prefusion and postfusion conformations [22].

### HMPV F trimer production and purification

We used transient transfection to express all HMPV F glycoproteins in Expi293F cells via transient transfection with ExpiFectamine 293 (Thermo Fisher) according to the manufacturer’s protocol. Cell culture supernatants were harvested after 5–6 days after transfection, and proteins were purified from the supernatants using tandem Ni^2+^ (Roche) and Streptactin (IBA) affinity purification. The C-terminal purification tags were removed by thrombin digestion at 4°C overnight, and proteins were further purified by size exclusion chromatography (SEC) with a Superose 6 Increase 10/300 GL (Cytiva) in PBS. The postfusion version of the F protein was designed by replacing the furin cleave site with six arginine residues, RRRRRR, and deleting the N-terminal fusion peptide. His tag and Strep tag were added at the C-terminus of the ectodomain of the F protein. The antigenicity of the postfusion protein was characterized with human antibodies MPF5 and MPE33 [22].

### Octet assessment

A fortéBio Octet Red384 instrument was used to measure binding kinetics of HMPV F variants to antibodies that target the prefusion or postfusion F form (MPE8, MPE33 and MPF5). All assays were performed with agitation set to 1,000 rpm at 30°C in phosphate-buffered saline (PBS) supplemented with 1% bovine serum albumin (BSA) to minimize nonspecific interactions. Ni-NTA sensor tips were equilibrated for 60 s in PBS + 1% BSA prior to loading HMPV F variants. Biosensor tips were then equilibrated for 60 s in PBS + 1% BSA prior to measuring association with antigen binding fragments (Fabs) in solution (500.0 nM to 7.8 nM) for 300 s; Fabs were then allowed to dissociate for 300 s. Data analysis and curve fitting were carried out using Octet software, version 9.0. Experimental data were fitted with the binding equations describing a 1:1 interaction. Global analysis of the data sets assuming reversible binding (full dissociation) was carried out using nonlinear least-squares fitting allowing a single set of binding parameters to be obtained simultaneously for all the concentrations used in each experiment.

### Thermal melting temperature (Tm) measurement

We measured thermal melting temperature (Tm) of selected HMPV F variants by differential scanning calorimetry (DSC) using a VP-DSC (GE Healthcare/MicroCal) and Nano differential scanning fluorimetry (NanoDSF, NanoTemper Technologies). The protein was diluted to 0.25 mg/ml in PBS and measured from 20 to 95°C at a rate of 1°C per minute. Tm were calculated from program provided by instruments.

### Negative stain-electron microscopy (EM)

Negative stain-EM was used to assess conformation of promising variants. Proteins were diluted to a concentration of 0.01–0.02 mg/ml with a buffer composed of 10 mM HEPES, pH 7, and 150 mM NaCl. A 4.7-μl drop of the diluted sample was placed on a glow-discharged carbon-coated copper grid for 15 s, and the drop of solution was removed with filter paper. The grid was washed three times with the same buffer, and adsorbed protein molecules were negatively stained with 0.7% uranyl formate. Datasets were collected at a nominal magnification of 100,000x (pixel size: 0.22 nm) using SerialEM [24] on an FEI Tecnai T20 electron microscope operated at 200 kV and equipped with an Eagle CCD camera, or at a nominal magnification of 57,000x (pixel size: 0.25 nm) on a Thermo Scientific Talos F200C electron microscope operated at 200 kV and equipped with a Ceta camera. Particle picking was performed automatically, and Relion 3.1 [25] was used for reference-free 2D classification.

### Production of single-chain FV of MPE8

A flexible linker (GGSGG x3) was used to connect VH and VL. The MPE8 single-chain Fv [VH-linker-VL] was codon optimized, synthesized and subcloned into VRC vector VRC8400. The His and Strep tags were appended to the C terminus with TEV protease cleave site. Antibodies were expressed in Expi293F cells via transient transfection with ExpiFectamine 293 (Thermo Fisher). Supernatants were harvested 6 days post-transfection, and purified using Nickel Sepharose Excel Resin (Cytiva), followed by SEC on a HiLoad Superdex 200 pg 16/600 column (Cytiva).

### Cryo-EM data collection, processing, and structure determination

Samples for cryo-EM grid preparation were produced by first mixing v3B_D12_D454C-V458C with MPE8 scFv at a molar ration 1:1.2 (monomeric F:scFv), purifying the complex by size exclusion chromatography and concentrating to 1 mg/ml. Purified complex was then diluted to 0.6 mg/ml in PBS and adjusted to have a final concentration of 0.005% (w/v) n-Dodecyl β-D-maltoside (DDM) to prevent preferred orientation and aggregation during vitrification. Cryo-EM grids were prepared by applying 3 μL of sample to a freshly glow discharged carbon-coated copper grid (CF 1.2/1.3 300 mesh). The sample was vitrified in liquid ethane using a Vitrobot Mark IV with a wait time of 30 s, a blot time of 3 s, and a blot force of zero. Cryo-EM data were collected using Leginon software [26] installed on a Titan Krios electron microscope operating at 300 kV, equipped with a Gatan K3-BioQuantum direct detection device. Exposures were taken with a total electron fluence of 58.06 e-/Å^2^. The total dose was fractionated for 2.5 s over 50 raw frames.

Cryo-EM data process workflow for the HMPV-scFv MPE8 complex, including motion correction, CTF estimation, particle picking and extraction, 2D classification, ab initio reconstruction, homogeneous refinement, heterogeneous refinement, non-uniform refinement, and local resolution estimation, were carried out with C3 symmetry in cryoSPARC 3.3 [27]. Using 192259 particles from 3D Ab-Initio classification and heterogeneous refinement, a final cryo-EM density map after sequential homogeneous and non-uniform refinements with overall resolution of 3.25 Å was obtained and used for iterative manual model building and real-space refinement in Coot [28] and in Phenix [29], respectively. The coordinates of HMPV F from PDB ID 7LZE and scFv MPE8 modeled with AlphaFold 2 [30] were used as initial models. Molprobity [31] was used to validate geometry and check structure quality at each iteration step. UCSF Chimera and ChimeraX were used for map fitting and manipulation [32].

### Mouse immunizations

All mouse experiments were reviewed and approved by the Animal Care and Use Committee of the Vaccine Research Center, National Institutes of Allergy and Infectious Diseases (NIAID), National Institutes of Health (NIH) and were housed and cared for in accordance with local, state, federal and institute policies within an Association for Assessment and Accreditation of Laboratory Animal Care (AAALAC) International accredited facility at the NIH. To assess the effectiveness of recombinant HMPV F trimer designs at eliciting neutralizing antibodies, groups of 10 CB6F1/J mice were immunized twice at weeks 0 and 3 intramuscularly via a needle syringe combination in the caudal thighs with 50 μg of recombinant HMPV F glycoprotein trimer designs combined with 10 μg Poly I:C and week 5 sera were assessed for autologous HMPV virus neutralization in vitro. Neutralizing antibody titers were determined using HMPV plaque-reduction neutralization assays.

### Rhesus macaque immunizations

The nonhuman Primate (NHP) Animal Study Proposal was reviewed and approved by the Animal Care and Use Committee of the Vaccine Research Center, NIAID, NIH and all animals were housed and cared for in accordance with local, state, federal and institute policies in an AAALAC International accredited facility at the NIH. Female and male Indian origin rhesus macaques with body weights between 2–9 kg were used for immunization studies. For each immunization, 1 ml of 100 μg immunogen mixed with 20% of Adjuplex, an adjuvant based on lecithin and carbomer homopolymer [33,34], in PBS, was injected via a needle syringe combination into the caudal thigh of each of the legs. Blood was collected two weeks post immunization for serological analyses.

### Virus neutralization

The neutralization assays were done using live HMPV based on a published method [35]. Briefly, sera were diluted at 1:10 with OptiMEM and inactivated at 56°C for 30 minutes. Serial four-fold dilutions of serum were made in 96-well plate and incubated with approximately 20–60 plaque forming units (PFU) per well of HMPV at 37°C for 60 minutes, and then transferred onto Vero cell monolayers in 24-well plates. HMPV a trypsin-dependent virus whose activation and replication require cleavage of the fusion protein. After cells and serum samples were incubated at 37°C for 60 minutes, and monolayers were washed to remove serum that contains trypsin inhibitor, and a trypsin-containing 1% methylcellulose overlay in Opti-MEM (Gibco) is added. After a 6-day incubation, the methylcellulose was removed, plates were fixed with 80% methanol, and plaques were visualized by immunostaining using rabbit hyperimmune serum made against sucrose gradient purified HMPV CAN97-83. The neutralizing titer, defined as the reciprocal of the test serum dilution at which the number of HMPV plaques was reduced by 60%, was calculated by linear regression analysis. Assay-to-assay variability can be induced from wash steps and trypsin digestion; therefore, it may not be appropriate to directly compare or rank the results of different assays.

### Flebogamma absorption assay

Absorption assays were conducted using a fortéBio Octet Red384 instrument. In brief, Flebogamma (10%, Lot#A4GLE00021, Grifols) was titrated with either prefusion or postfusion immunogen after the removal of the purification 6xHis tag. A series of 2-fold dilutions of prefusion immunogen (HMPV: HMPV v3B_Δ 12 D454C-V458C; RSV [15]: RSV DS-Cav1; PIV3 [17]: PIV3 GCN4, A464V, I474Y) or postF [15–17] starting at a concentration of 512 μg/mL was mixed with Flebogamma. After a 30-minute incubation, His-tagged HMPV prefusion and postfusion F at a concentration of 20 μg/mL in 1% BSA/PBS were loaded onto NTA biosensors for 300 s. Sensor tips were then equilibrated in 1% BSA/PBS for 60 s, followed by dipping into the Flebogamma mixture for 300 s, and a subsequent dissociation step for an additional 60 s. The responses after association were plotted against the titrant concentration. Triplicate repeats were performed to calculate the deviation. RSV F proteins were antigenically characterized using antibody D25 and MPE8, while PIV3 fusion protein was characterized with antibody PIA174 as reported before [16,17,22].

## Results

### Optimization of F2-F1 linkage improves yield and antigenicity of HMPV F trimer in the pre-F state

**Fig 1:**
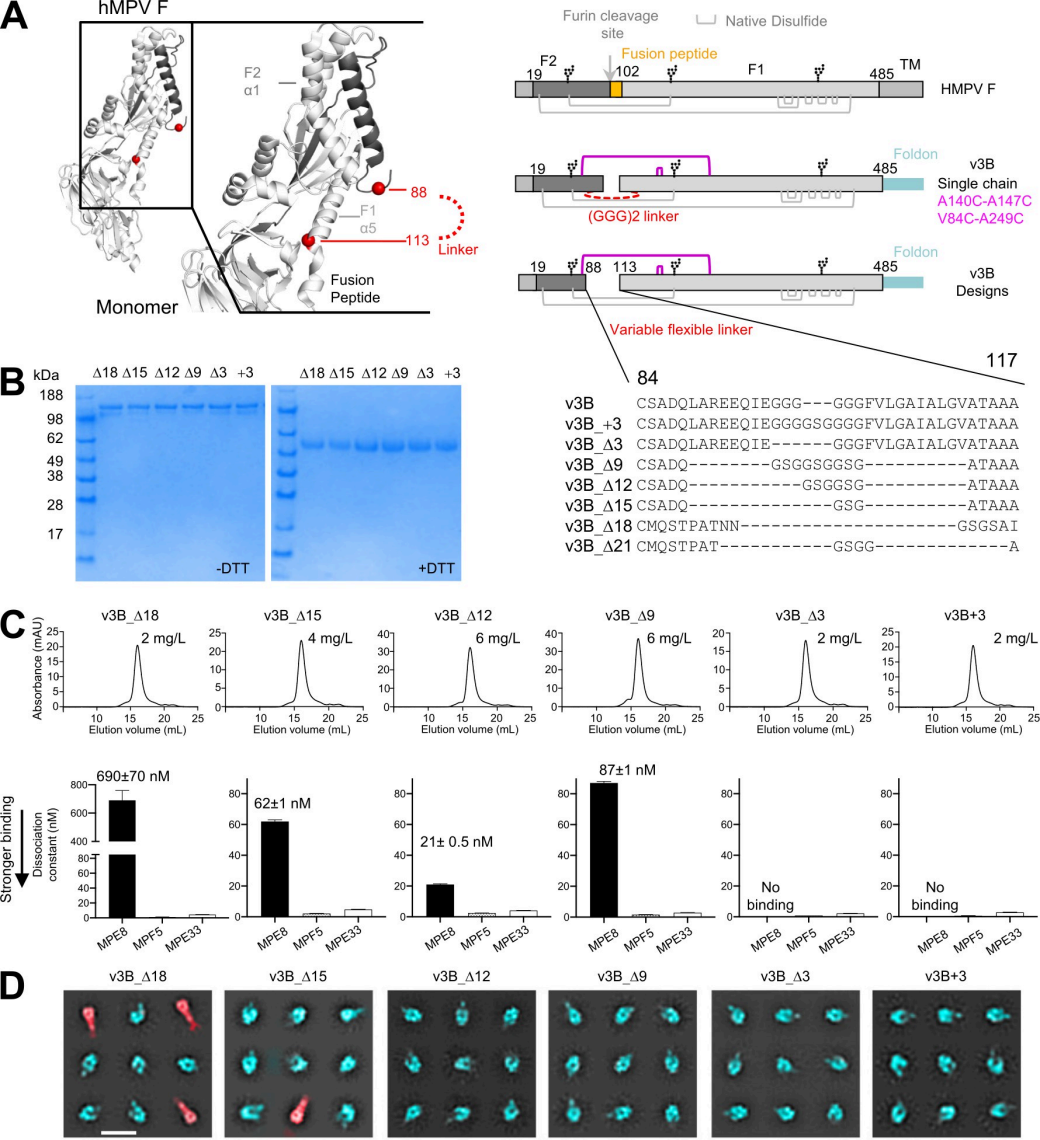
Modification of F2-F1 linkage further stabilize HMPV F trimer in a prefusion state. (**A**) Structure-based design of a single chain pre-F HMPV F based on PDB ID 5WB0; additional designs are shown in S1 Table; D is length change compared to the v3B. (**B**) SDS-PAGE analysis of interprotomer disulfide-stabilized HMPV F prefusion variants; (**C**) Analysis of single chain HMPV F prefusion variants by size exclusion chromatography with yields (top row) and immunogen binding affinity to antibodies (bottom row); (**D**) Negative-stain electron micrographs of v3B single chain variants. 2D classes indicate that most trimers are in prefusion conformation which lack the long tail (pseudo-colored in cyan), whereas some F trimers are in postfusion conformation which appear as elongated torpedo shape with a tail (pseudo-colored in red). Scale bar = 200 Å.

Based on the pre-F structure of trimeric HMPV F [21], we previously designed an HMPV F variant (v3B) with an intraprotomer disulfide bond (A140C-A147C) as well as an interprotomer disulfide (V84C-A249C). Although this interprotomer disulfide-stabilized HMPV F trimers could elicit significantly higher neutralizing responses in mice and macaques [22], v3B expression was suboptimal, and v3B was not recognized by the pre-F specific antibody MPE8 [23], indicating its antigenicity to be also suboptimal. To test if optimization of the connection between the two subunits (F1 and F2) of HMPV F could improve yield and antigenicity, we engineered HMPV F variants with different length of the F2-to-F1 linker and different positions for the connection (Fig 1A and S1 Table).

In Expi293 cells, six of these constructs expressed at sufficient levels to allow characterization of their physical and antigenic properties. SDS-PAGE analysis indicated interprotomer disulfides to be formed in all six variants, as judged by the presence of a higher molecular weight band, which correspond to a covalent-linked trimer in the absence of reducing agent (Fig 1B). Antigenic analysis by biolayer interferometry (BLI) indicated all six variants to be recognized by antibodies MPE33 and MPF5, which recognized both pre-F and post-F conformations with similar affinity; recognition by the pre-F specific antibody, MPE8, appeared to be sensitive to the length of the F2-F1 linker (Figs 1C and S1). Of these, variant v3B_Δ12, comprising a GSGGSG linker between residues 89 and 112, showed greater than 10-fold enhanced expression and a K_D_ of 21 nM to MPE8 (Fig 1C). Both differential scanning calorimetry (DSC) and differential scanning fluorimetry (DSF) (S2 Fig) showed variant v3B_Δ12 to be the most stable, with an increase in melting temperature (Tm) of ~3°C. Importantly, negative stain-electron microscopy (EM) analysis (Fig 1D) revealed that post-F conformation with elongated long was still present in some variants (v3B_Δ18, v3B_Δ21), while of v3B_Δ12 indicated high prevalence of the pre-F conformation (Fig 1D). Therefore, we chose this construct for further development.

### Additional DS and Pro mutations can further stabilize HMPV F trimer in a prefusion state

**Fig 2:**
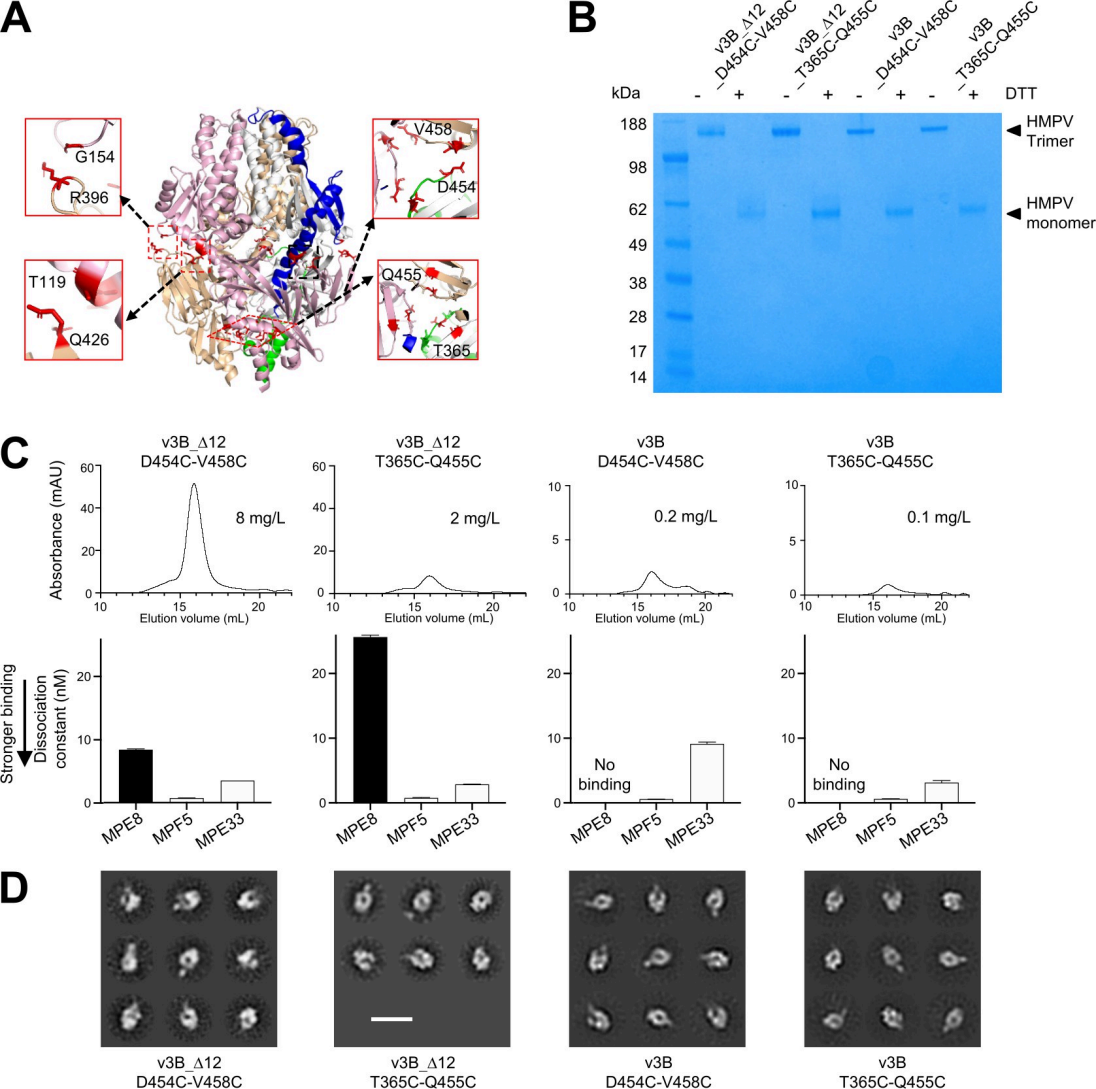
Additional disulfide-bonds can stabilize HMPV F trimer in a prefusion state. (**A**) Structure-based design of interprotomer disulfides based on the prefusion structure of HMPV F (PDB ID 5WB0); additional designs are shown in S1 Table. (**B**) Properties of HMPV Fs. SDS-PAGE analysis of interprotomer disulfide-stabilized HMPV F prefusion variants. (**C**) Analysis of HMPV F prefusion variants by size exclusion chromatography SEC and expression level (top row) and binding affinity (bottom row) of HMPV designs with additional disulfide bonds. (**D**) Negative-stain electron micrographs of HMPV F trimer variants. 2D classes indicate the F proteins are in prefusion conformation. Scale bar = 200 Å.

We analyzed the structure of trimeric pre-F HMPV F (PDB: 5WB0) [21] for positions where additional disulfide linkages could be introduced (Fig 2A). We synthesized 12 variants of v3B_Δ12 with additional disulfides (S1 Table) and assessed their expression. Only two of these constructs expressed at levels that permitted characterization by analytical size exclusion column and physical properties (Fig 2B and 2C). Yields for these two were substantially reduced in variants without the optimized F2-F1 linker (Fig 2C).

Introduction of the cysteines D454C and V458C to v3B_Δ12 increased both expression as well as affinity to MPE8 (Fig 2C). Introduction of cysteines T365C and Q455C reduced expression yields from 6 to 2 mg/L, did not alter recognition by MPE8 (Fig 2C), but did increase Tm by 5°C as assessed by DSF (S2 Fig). Negative stain-electron micrographs demonstrated that the purified variants with additional disulfides were in trimeric forms that appeared to be primarily in the pre-F conformation (Fig 2D).

**Fig 3:**
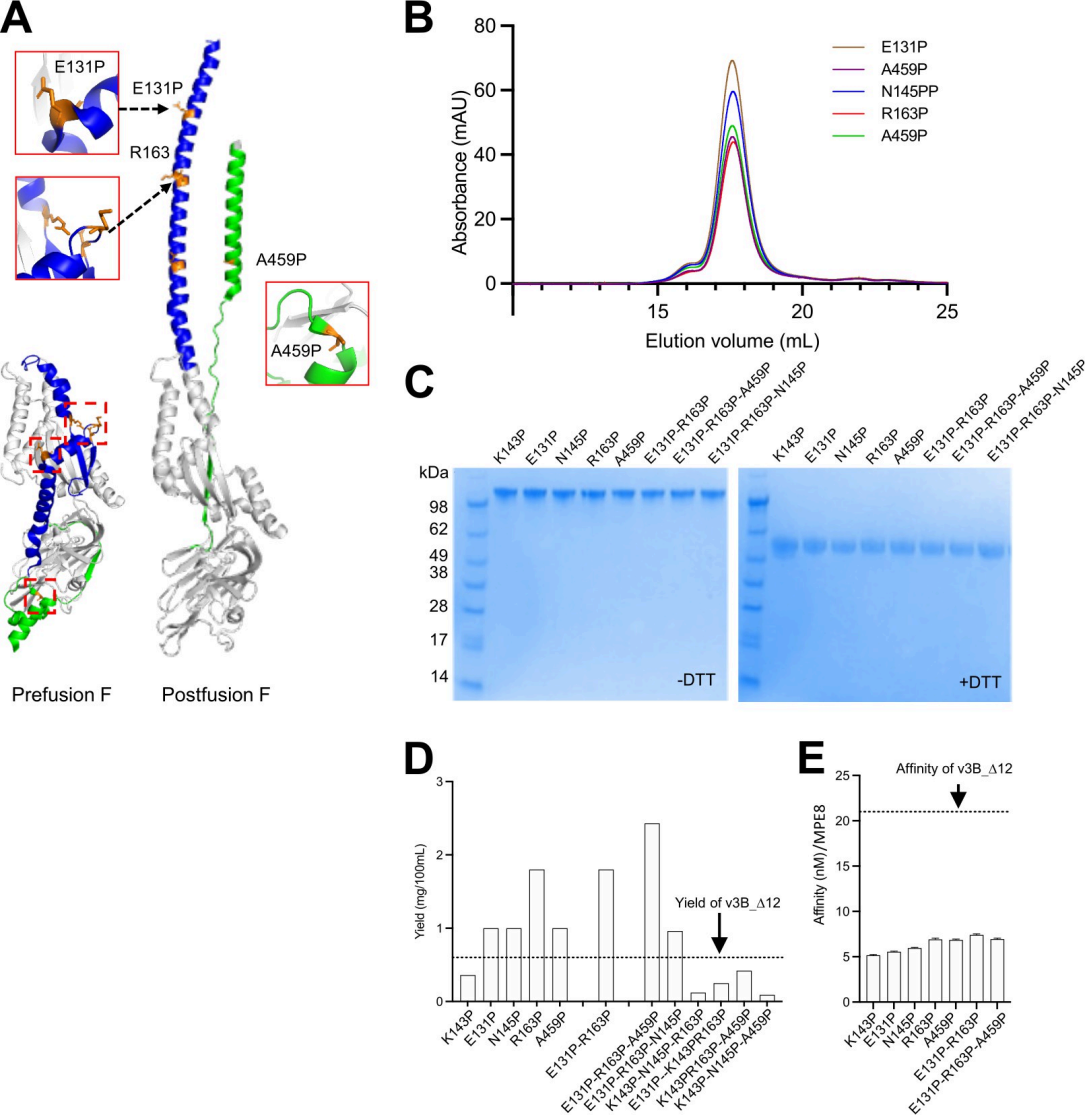
Pro mutations can increase yield and enhance recognition by the prefusion specific antibody, MPE8. (A) Structure-based design of proline substitutions based on prefusion-postfusion conformation change structure of HMPV F (PDB ID 5WB0) (B) Analysis interprotomer disulfide-stabilized HMPV F prefusion variants by size exclusion chromatography (C) SDS-PAGE analysis of interprotomer disulfide-stabilized HMPV F prefusion variants. (D) Additional proline substitutions increase the expression yield of HMPV F, the yield of v3B_D12 is show as dotted line. (E) The binding affinity of HMPV designs to MPE8, the affinity of v3B_D12 is show as dotted line.

Proline substitution is another widely used strategy to stabilize conformations of type 1 fusion machines [36–39], and a single appropriately placed proline is able to stabilize the pre-F conformation of HMPV F [21]. We analyzed the pre-F structure of HMPV F for residues that moved more than 5 Å between prefusion and postfusion conformations, and introduced Pro substitutions at five sites: E131, K143, N145, R163 and A459. All five of these variants, each with a single Pro substitution, showed improved recognition by MPE8 (Fig 3E and S2 Table). Four of the variants showed improved expression yield for (Fig 3D), with the best expressed variant, R163P, increasing yield by a factor of three. We also tested combinations of proline substitutions. Variants with the two proline substitutions K143P and N145P showed reduced expression yields (Fig 3D); variants comprising combination of the other three proline mutations (E131P, R163P and A459P; 3P) showed substantially increased yields (Fig 3D).

Thermal stability analysis by nano differential scanning fluorimetry verified the thermostability of the HMPV F variants with additional disulfides or proline mutations (S3 Fig). To evaluate long-term stability, we selected the immunogens stabilized with disulfide and 3P mutations and incubated at 37°C. SDS-PAGE and antigenic analysis revealed that stabilized immunogens still retained 60–80% prefusion antigenic stability after six months, indicating good long-term stability (S3C Fig).

### Structure of variant v3B_Δ12_D454C-V458C with MPE8

**Fig 4:**
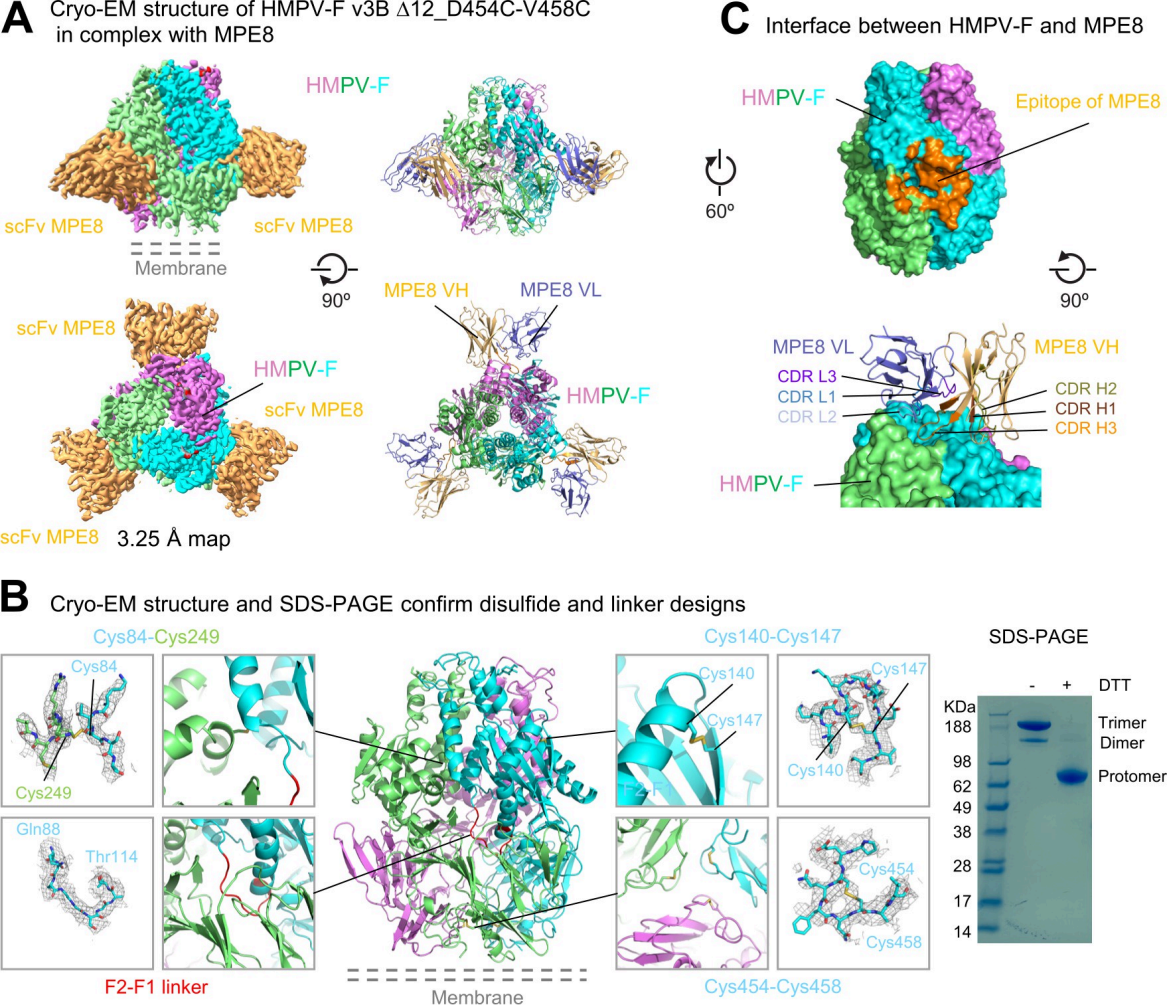
Cryo-EM structure of prefusion-stabilized HMPV F with MPE8-variable domain (Fv) provides details of prefusion structure and of disulfide bonds formation. (**A**) Structure of HMPV F trimer (v3B Δ12_D454C-V458C) with single chain MPE8 Fv. Cryo-EM density map and fitted coordinates of HMPV F protomers were colored in cyan, violet and lime, respectively. The density of the scFV of MPE8 was colored in orange (left), while the VH and VL coordinates were colored orange and slate (right). (**B**) Cryo-EM structure confirms design of intra-chain disulfide bonds and linker between F1 and F2. Each design and its observed EM density map were shown in zoom-in boxes. Density maps were contoured at 1.5 to 4σ in gray meshes. Even though the density for the interchain disulfide bond between Cys84 and Cys249 was not clear, SDS-PAGE indicated single chain trimeric F converted monomeric form in the presence of reducing agent DTT, confirming the formation of interchain disulfide bond (right). Molecular weight marker was run alongside the HMPV F samples. (**C**) Interactions between HMPV F and MPE8. The epitope of MPE8 located at the interface between two HMPV F protomers is colored orange (top), CDR H3 of MPE8 reaches into the shallow depression at the protomer junction.

While SDS-PAGE confirmed the formation of interprotomer disulfide bonds, the formation of intraprotomer disulfides was unclear, and we therefore sought to obtain structural information. Attempts to obtain the cryogenic electron-microscopy (cryo-EM) structure of v3B_Δ12_D454C-V458C with the MPE8 antigen-binding fragment (Fab) failed because of Fab-Fab interactions that caused the formation of ordered arrays on cryo-EM grids. Substitution of the MPE8 Fab with the single-chain variable fragment (scFv) lead to dispersed particles, with reconstruction density at 3.25 Å resolution (Figs 4A, S5, and S6 and S3 Table).

Clear electron density between Cys140-Cys147 and between Cys454-Cys458 confirmed the formation of these intraprotomer disulfide bonds (Fig 4B). The reconstruction also provided clear density for the optimized linker between F2 and F1 subunits (Fig 4B, left). While electron density between the potential interprotomer disulfide linking Cys84-Cys249 was broken; SDS-PAGE analysis, however, demonstrated the formation of interprotomer disulfides (Fig 4B, right). A smaller band with a size of a dimer was visible below the trimer band, suggesting that interprotomer disulfide bonds formed between some neighboring protomers but did not form between others.

MPE8 bound a quaternary epitope at the surface of the trimer to residues from two adjoining HMPV F protomers with similar mode of approach to that in the RSV F-MPE8 complex [40]. The interface buried ~1280 Å^2^ of epitope surface that spanned over two neighboring protomers with ~75% of the surface provided by the DI and DIII domains of the major binding protomer and ~25% of the surface from the DII domain of the neighboring protomer (Fig 4C). All complementarity-determining regions (CDR) from both heavy and light chain were involved in binding HMPV F. While CDRs H1, H2, L1 and L3 contacted only the major binding subunit, the 15-residue long CDR H3 reached into a pocket at the inter-protomer junction making extensive contacts to both protomers with its >500 Å^2^ binding surface. Additional binding to the neighboring protomer was provided by CDR L2 (Fig 4C), therefore, interactions from CDR H3 and CDR L2 stabilized the HMPV F into its prefusion conformation.

To compare the conformation of MPE8-bound HMPV F that were stabilized by different approaches, we first superposed our MPE8-HMPV F complex with a recently deposited HMPV F structure [41] (PDB ID: 8CW9) over the MPE8 heavy chain Fv region and then analyzed conformational difference between the MPE8-bound protomers. While the major binding protomer showed an overall root-mean-square deviation (RMSD) of 2.1 Å over the Cα-atoms of 400 F2 and F1 residues, the quaternary epitope of MPE8 showed much less deviation: for example, the central strands of HRA (residues 146 to 161), the helix-turn-helix motif (residues 223 to 248), the β11/β12 hairpin (residues 326:336) and the domain II region (residues 371 to 432) had only Cα-atom RMSDs of 0.35 Å, 0.42 Å, 0.27 Å and 0.61 Å, respectively, suggesting that MPE8 could firmly stabilize the quaternary epitope. Despite different designs between the HMPV F compared, especially one with a cleavage site between F2 and F1 and the other with a non-cleavable single chain linker connecting F2 and F1, the regions flanking the single chain linker (F2 C-term helix and the F1 N-term helix) only displayed a Cα-atom RMSDs of 0.53 Å between these two HMPV F structure, indicating that the F2-to-F1 linker preserved the HMPV F with high fidelity.

### Prefusion stabilized HMPV F variants induce high-titer neutralizing responses in mice

**Fig 5:**
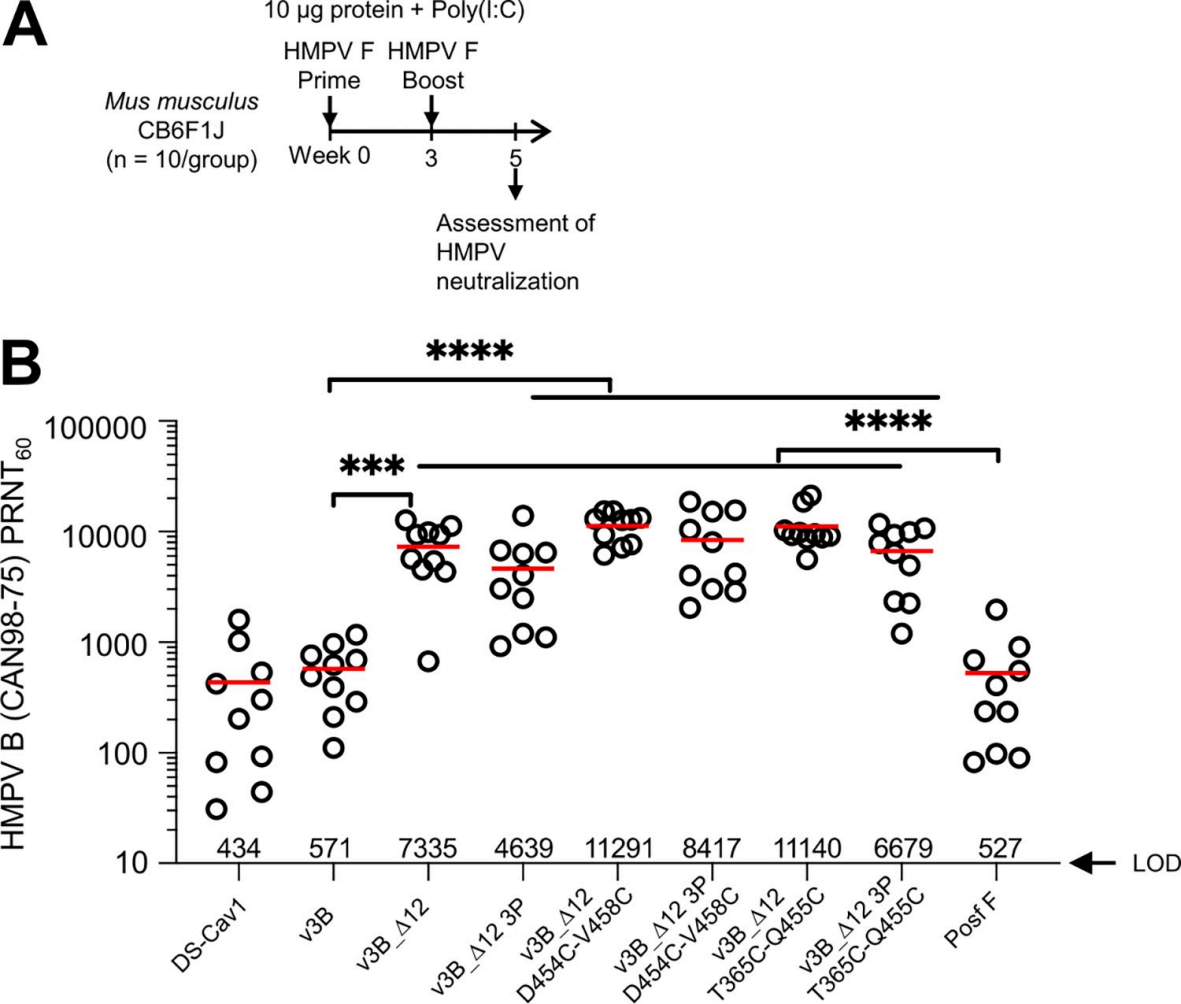
Prefusion-stabilized HMPV F variants induce high titer neutralizing responses in mice. (**A**) Immunization regimen for CB6F1J mice (n = 10 /group) with two HMPV F immunizations followed by serum analysis at week 5. (**B**) Elicited HMPV F neutralization by prefusion F immunogens. Limit of detection (LOD) is shown as an arrow.

Three prefusion immunogens (v3B_Δ12, v3B_Δ12_D454C-V458C, and v3B_Δ12_T365C-Q455C) as well as these same variants with additional 3P mutations were assessed for their ability to elicit HMPV-neutralizing responses in naïve mice. We immunized CB6F1/J mice with 10 μg doses of each of the HMPV F glycoprotein variants combined with 50 μg polyinosinic-polycytidylic acid (poly-I:C) adjuvant at weeks 0 and 3 and measured the functional activity of week 5 sera in a plaque reduction neutralization assay (PRNT) using the homologous HMPV B2 strain. The optimized single chain variants (v3B_Δ12, v3B_Δ12_D454C-V458C, and v3B_Δ12_T365C-Q455C) elicited especially high neutralizing titers, averaging over 11,000, 20-fold higher than elicited by post-F and 1.5-fold higher than those elicited by v3B_Δ12 (Fig 5B). Notably, non-3P–containing immunogens induced significantly higher HMPV-neutralizing titers than 3P-containing immunogens (P = 0.006) (S7 Fig). Overall, the single chain triple-disulfide stabilized pre-F HMPV F variants with Pro substitution at residue 185 that preserved MPE8 antigenicity elicited the highest neutralization titers in mice, with v3B_Δ12_D454C-V458C inducing titers of 11,291 by week 5.

### Assessment of immunity recall in pre-exposed NHPs

**Fig 6:**
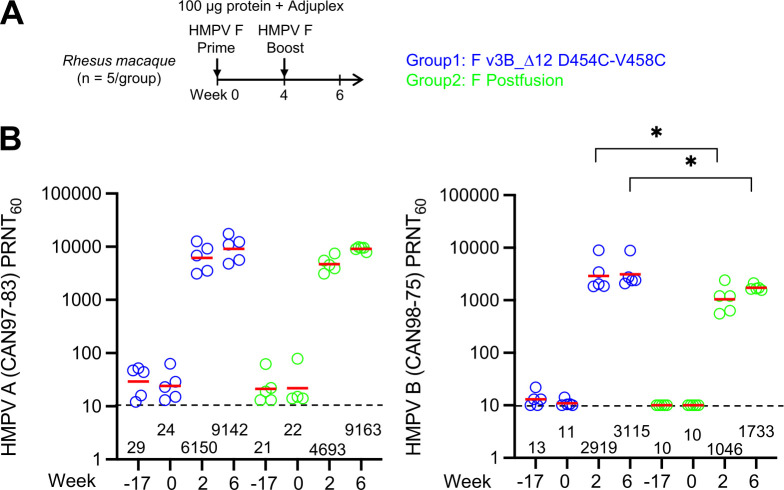
Immunization of rhesus macaques shows interprotomer disulfide-stabilized variants of either prefusion or postfusion HMPV F induce neutralizing responses many times the average titer in healthy adult humans. (**A**) Immunization regimen for rhesus (n = 5/group) with two HMPV F immunizations followed by serum analysis at week 6. (**B**) Neutralizing responses graphed with geometric mean titers provided. Limit of detection is shown as a dotted line.

In addition to vaccination of infants, who are naïve to HMPV, other target vaccine populations include the elderly, who have likely been previously exposed to HMPV. To assess the ability of the v3B_Δ12_D454C-V458C trimer to recall neutralization, we chose to assess in rhesus macaques, who are known to be infected by HMPV [42,43]. We assessed rhesus macaques for those with ELISA-positivity to F, but with responses too low to be observed by biolayer interferometry (BLI) (S8 Fig), and found 10 animals, which we randomized into two groups of 5 each. One group was immunized with v3B_Δ12_D454C-V458C trimer and the other group with HMPV post-F, 100 μg dose of glycoprotein combined with the adjuvant Adjuplex, at weeks 0 and 4. We measured the functional activity of week 2 and 6 sera in a PRNT using both the homologous HMPV B2 strain and a heterologous A2 strain (Fig 6A and S4 Table). Notably, in macaques immunized with v3B_Δ12_D454C-V458C, A2 titers of 6,150 were observed at week 2, which rose to 9,142 by week 6. B2 strain titers were lower, but still high, 2,919 at week 2 and 3,115 at week 6 (Fig 6B). We also evaluated the binding of individual NHP serum samples to HMPV F prefusion protein v3B_Δ12_D454C-V458C and postfusion protein by ELISA and Octet (S9 Fig). Our results showed that the group immunized with v3B_Δ12_D454C-V458C exhibited higher ratios of prefusion to postfusion F antibody binding, although the difference was not statistically significant (S9A and S9B Fig). In the naïve mouse study, we observed a significant increase in the ratio of prefusion to postfusion (S9C Fig).

We observed responses to post-F to also be high, suggesting that the recall response may be less discriminating, in terms of its being triggered by a specific immunogen. We note in this context that immunization with RSV F DS-Cav1 does not induce HMPV neutralizing titers in naïve mice but can induce HMPV-neutralizing responses in pre-exposed adults as reported by Phung and colleagues [44].

### Human globulin adsorption analysis

**Fig 7:**
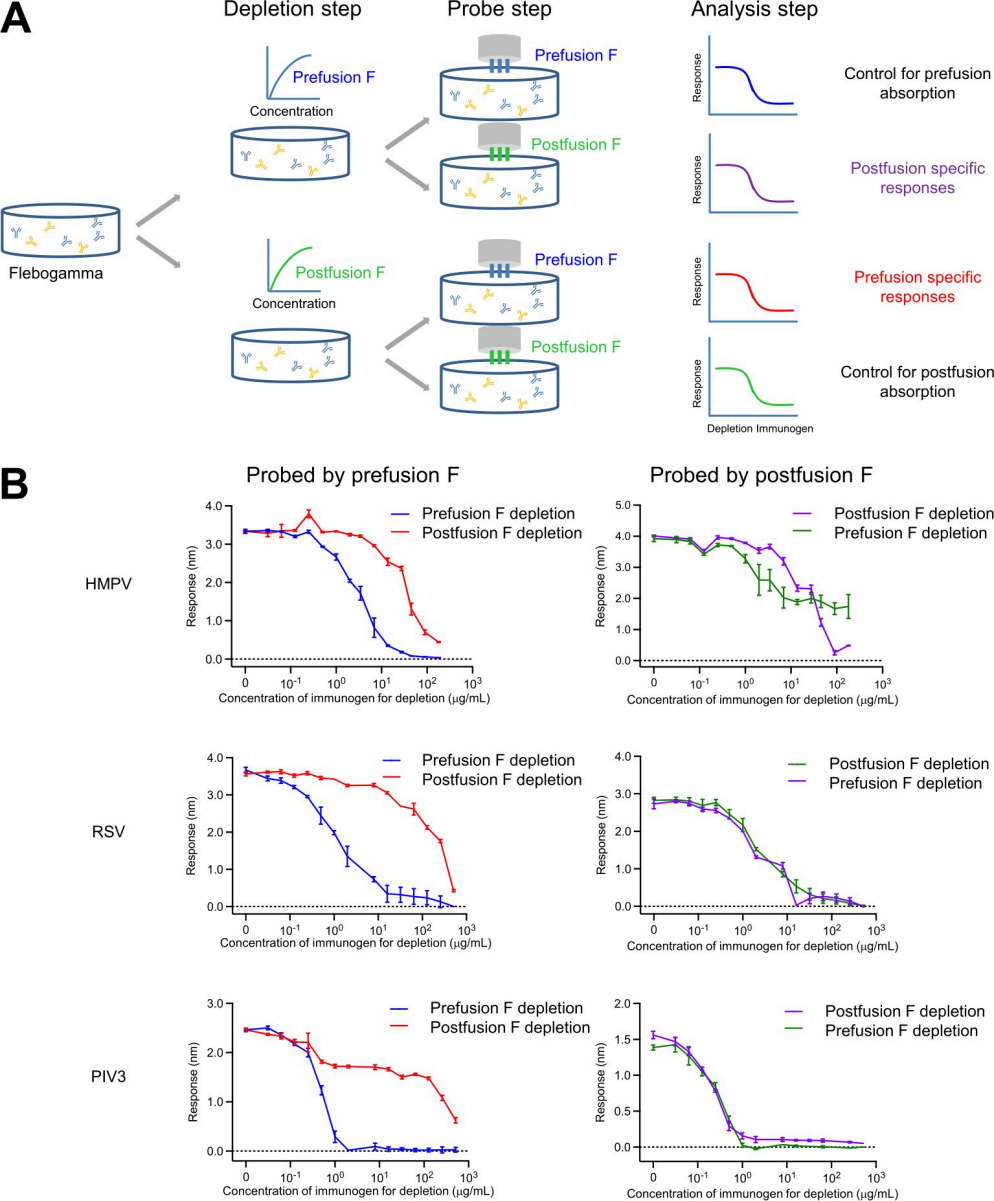
Human globulin analysis indicates prefusion conformation stabilized F glycoprotein absorbs neutralizing responses more efficiently than the postfusion form. (**A**) Experimental scheme for the depletion of Flebogamma. (**B**) Depletion of Flebogamma with prefusion- and postfusion-stabilized HMPV(top panel), RSV (middle panel), and PIV3 F (bottom panel) glycoproteins.

Adsorption analysis provides another means to assess the v3B_Δ12_D454C-V458C immunogen. In this type of analysis, antigens are assessed for their ability to adsorb antibodies in serum as well as for their ability to detect what remains after adsorption. Previous analysis with a first-generation single proline-stabilized HMPV F trimer of human Flebogamma, pooled human globulin used for treatment of primary immunodeficiency, found this first-generation HMPV F trimer to deplete about the same titers as post-F of HMPV [21]. Here, we extended our analysis to include pre-F and post-F forms of HMPV, RSV and PIV3, probing pre-F and post-F specific responses after removing antibodies from Flebogama through titrating either with trimers of post-F or pre-F-stabilized prefusion F (Fig 7).

Detecting with pre-F after depletion with either pre-F or post-F, however, we observed substantial depletion at two-to-three orders of magnitude lower amounts of pre-F than with post-F for RSV and PIV3, but for HMPV, only one order of magnitude lower. These observations suggest there to be substantially more postfusion F responses that cross-react with pre-F from HMPV than with pre-F from RSV or PIV3. Detecting with post-F, we observed both pre-F and post-F to deplete similarly with RSV and PIV3, while with HMPV, more titers remained after pre-F adsorption, indicating higher levels of post-F-specific responses against HMPV than for RSV and PIV3. Overall, these adsorption results indicate substantially more post-F responses for HMPV than RSV and PIV3; the triple disulfide-stabilized pre-F v3B_Δ12_D454C-V458C, nonetheless, adsorbed HMPV-directed responses more fully than post-F.

## Discussion

With structure-based design, it is critical to know the appropriate target shape to fix or to stabilize. Initial immunogenicity results with the HMPV F revealed the immunogenicity of first-generation pre-F and post-F [21] as well as of interprotomer disulfide-stabilized variants [22] to be similar. Theoretically, however, neutralizing epitopes on post-F should all be present on pre-F–with pre-F being a more comprehensive repository of neutralizing epitopes, with additional epitopes not present on post-F. Here, we investigate the impact of further stabilizing pre-F, developing a single-chain, triple-disulfide-stabilize variant, which elicited high titer neutralizing responses in both naïve mice (after two injections) and pre-exposed macaques (after a single injection).

A recent publication suggests processed pre-F HMPV trimers with a Pro mutation to be superior to uncleaved trimer in terms of thermal stability (62.4°C versus 55.5°C), shedding light on the significance of the F2-F1 linker between HMPV subunits to pre-F thermal stability [45]. Relevant to this, our series of linker designs indicates that altering the length of the linker can have a significant impact on the thermal stability of the HMPV protein–and we further show that the linker also substantially impacts immunogenicity.

In naïve mice, we observed elicited titers from disulfide-stabilized variants of pre-F to be significantly higher than those elicited by post-F (Fig 5). Recently, Hsieh and colleagues also reported the development of disulfide-stabilized pre-F trimers [46], which elicited higher neutralizing titers than a post-F. Whether the higher observed titers resulted from greater fixation in the pre-F shape, alterations in immunogenicity related to alterations in flexibility imparted by disulfide stabilization, or a combination of these two effects is unclear. Perhaps relevant to this, we did observe increases in the recognition by the pre-F specific antibody, MPE8, to correlate with increased neutralizing (S7 Fig), indicating that at least some of the increase does relate to increased fixation in the pre-F state.

In phase III trials of recently approved RSV vaccines, a single immunization of prefusion-stabilized F glycoprotein prevents RSV-associated lower respiratory tract illness in adults [47–49]. Similar to this observation in the RSV trials, we observed that a single immunization of HMPV F immunogen elicited over 100-fold higher recall responses in rhesus macaques (Fig 6). However, we also observed that both pre-F and post-F HMPV immunogens elicited similar recall neutralizing responses. One possible explanation is that both pre-F and post-F conformations share substantial amount of neutralizing epitopes, as shown in the adsorption analysis (Fig 7). On the other hand, for infants and babies who are not pre-exposed to HMPV, responses will likely be more similar to those that we observed in naïve animals, and thus a prefusion-stabilized HMPV F vaccine will likely induce stronger response and better protection, though this remains to be confirmed clinically.

## Conclusion

The triple-disulfide stabilized pre-F immunogen, v3B_Δ12_D454C-V458C, appears to be a promising vaccine immunogen. It will be critical, however, to assess this immunogen in appropriate target populations. Almost all humans are infected with HMPV by the age of five [50–52], suggesting that vaccinations targeting a naïve population will need to be carried out early in life. Our results in naïve mice indicate two immunizations with v3B_Δ12_D454C-V458C to elicit high titer neutralizing responses (Fig 5). Otherwise, the target vaccine population will be the pre-exposed. Our results in pre-exposed rhesus macaques indicate high titer neutralizing responses, after a single immunization with v3B_Δ12_D454C-V458C or postF (Fig 6). It will be interesting to see if v3B_Δ12_D454C-V458C can induce high titer responses in humans, whether delivered as a protein subunit vaccine with adjuvant as we have done here or delivered as a lipid-encapsulated mRNA-encoded vaccine, as has been done for SARS-CoV-2 [53,54] and is being tested clinically with other pre-F immunogens such as those for RSV [55]. Perhaps relevant to this, we note that additional disulfide stabilization did appear to improve the immunogenicity elicited by pre-F stabilized RSV F [16], and such stabilization appeared to be crucial in eliciting high titer responses with mRNA-encoded vaccines [55–58].

## Supporting information

S1 Table Engineered HMPV F glycoprotein variants. (PDF)

S2 Table Binding affinity of HMPV designs with additional disulfide bonds and proline substitutions. (PDF)

S3 Table Cryo-EM Data Collection, Refinement and Validation Statistics for HMPV F in complex with scFv MPE8. (PDF)

S4 Table Serum neutralization of HMPV subtype A2 and B2 strain. (PDF)

S1 Fig Octet Bio-Layer interferometry profiles for binding of MPE8, MPF5 and MPE33 Fabs to HMPV prefusion-stabilized F variants. (TIF)

S2 Fig Thermal stability of HMPV F variants. The melting temperature of variants was determined by (**A**) Nano Differential Scanning Fluorimetry (NanoDSF) and (**B**) Differential scanning calorimetry (DSC). (**C**) Summary of melting temperature of HMPV F variants.
(TIF)

S3 Fig Thermal stability of HMPV F variants by Nano Differential Scanning Fluorimetry (NanoDSF). Variants with proline substitution were shown in panel (**A**) and variants with disulfide bonds were shown in (**B**). (**C**) SDS-PAGE and antigenic analysis of disulfide and 3P stabilized HMPV F variants after incubation at 37°C for the indicated time.
(TIF)

S4 Fig Negative-stain electron micrographs of HMPV F trimer variants with Proline substitution. (TIF)

S5 Fig Cryo-EM data processing workflow leading to the structure of HMPV F in complex with variable domains of antibody MPE8. (TIF)

S6 Fig Cryo-EM details of the HMPV F in complex with scFv MPE8. **(A)** Representative micrograph of HMPV F in complex with scFv MPE8. (**B**) Representative 2D class averages are shown. (**C**) The gold-standard Fourier shell correlation resulted in a resolution of 3.25 Å for the overall map using non-uniform refinement with C3 symmetry (left panel); the orientations of all particles used in the final refinement are shown as a heatmap (right panel). (**D**) The local resolution of the final overall map is shown contoured at 0.448 (5.6s). Resolution estimation was generated through cryoSPARC using an FSC cutoff of 0.5. (**E**) Representative density is shown for the interface between CDR H3 and HMPV F. The contour level is 1.5σ.
(TIF)

S7 Fig Analysis of HMPV F elicited responses in mice. (**A**) Correlation of antigenicity and immunogenicity of HMPV F variants. (**B**) Comparison of titers elicited by single chain prefusion stabilized with and without 3P mutations.
(TIF)

S8 Fig Selection of NHPs with pre-exposed titers against HMPV F. (**A**) Pre-exposed titers were measured using pre-bleeding by ELISA against HMPV F prefusion (HMPV-F v3B D12_D454C-V458C) and postfusion(postF) immunogens. Neutralization titer were measured by plaque reduction neutralization tests (PRNT) using HMPV subtype A and B strain. 10 NHPs highlighted as color red were selected for HMPV F immunization. (**B**) Measured anti-F ELISA titer is corelated with neutralization titers. (**C**) 10 selected NHPs were assigned to prefusion group (blue) and postfusion group (green); ELISA IgG responses with area under curve (AUC) and endpoint titers of each group was shown. Pre-exposed titers against RSV were measured by ELISA with IgG endpoint titers against RSV F DS-Cav1 (right panel).
(TIF)

S9 Fig Serological analysis of HMPV F immunized animals. **(A)** Anti-HMPV prefusion and postfusion IgG responses of NHP sera were measured by ELISA against HMPV F prefusion or postfusion immunogens. Ratios of prefusion vs postfusion were in right panel. (**B**) Anti-HMPV prefusion and postfusion responses of NHP sera were probed by Octet. (**C**) Three mouse study groups were selected, and anti-HMPV prefusion and postfusion responses were probed by Octet.
(TIF)
