# Supplementary material for: Structure-based design of a single-chain triple-disulfide-stabilized fusion-glycoprotein trimer that elicits high-titer neutralizing responses against human metapneumovirus
Source: PLoS Pathog. 2023 Sep 22;19(9):e1011584. doi: 10.1371/journal.ppat.1011584 (PMC10516418; doi:10.1371/journal.ppat.1011584)
Supplement: S1 Table — (PDF) [file ppat.1011584.s001.pdf]

S1 Table. Engineered HMPV F glycoprotein variants.

Rationale	Name	Mutation	F1-F2 Linker residues	Linker sequence
Additional DS	v3B_G154C-R396C	A140C, A147C, A249C, V84C, G154C, R396C, 185P	97-103	GGGGGG
	v3B_GV154Cg-R396C	A140C, A147C, A249C, V84C, GV154Cg, R396C, 185P	97-103	GGGGGG
	v3B_G154CG-R396C	A140C, A147C, A249C, V84C, G154CG, R396C, 185P	97-103	GGGGGG
	v3B_D454C-V458C	A140C, A147C, A249C, V84C, D454C, V458C, 185P	97-103	GGGGGG
	v3B_L141C-A161C	A140C, A147C, A249C, V84C, L141C, A161C, 185P	97-103	GGGGGG
	v3B_E26C-G439C	A140C, A147C, A249C, V84C, E26C, G439C, 185P	97-103	GGGGGG
	v3B_T45C-V157C	A140C, A147C, A249C, V84C, T45C, V157C, 185P	97-103	GGGGGG
	v3B_E51C-K166C	A140C, A147C, A249C, V84C, E51C, K166C, 185P	97-103	GGGGGG
	v3B_E80C-D224C	A140C, A147C, A249C, V84C, E80C, D224C, 185P	97-103	GGGGGG
	v3B_A86C-G212C	A140C, A147C, A249C, V84C, A86C, G212C, 185P	97-103	GGGGGG
	v3B_F103C-G366C	A140C, A147C, A249C, V84C, F103C, G366C, 185P	97-103	GGGGGG
	v3B_G106C-P321C	A140C, A147C, A249C, V84C, G106C, P321C, 185P	97-103	GGGGGG
	v3B_T365C-Q455C	A140C, A147C, A249C, V84C, T365C, Q455C, 185P	97-103	GGGGGG
	v3B_S293C-S443C	A140C, A147C, A249C, V84C, S293C, S443C, 185P	97-103	GGGGGG
Modify the F2-F1 link region	v3B_Δ3	A140C, A147C, A249C, V84C, 185P	89-112	GGG
	v3B_Δ15	A140C, A147C, A249C, V84C, 185P	89-112	GSG
	v3B_Δ12	A140C, A147C, A249C, V84C, 185P	89-112	GSGGSG
	v3B_Δ9	A140C, A147C, A249C, V84C, 185P	89-112	GSGGSGGSG
	v3B_Δ21	A140C, A147C, A249C, V84C, 185P	85-112	MQSTPATGSGS
	v3B_+3	A140C, A147C, A249C, V84C, 185P	97-112	GGGgsgGGG
	v3B_L3c	A140C, A147C, A249C, V84C, 185P	95-112	LEVLFQGPgg
	v3B_Δ18	A140C, A147C, A249C, V84C, 185P	89-112	MQSTPATNNGSGS
	v3B_L4c _Δ22	A140C, A147C, A249C, V84C, 185P	89-112	MQLEVLFQGPgs
Extra IPDS to the v3B Δ12	v3B_Δ12_A120C-Q426C	A140C, A147C, A249C, V84C, 185P, A120C, Q426C	89-112	GSGGSG
	v3B_Δ12_T119C-Q426C	A140C, A147C, A249C, V84C, 185P, T119C, Q426C	89-112	GSGGSG
	v3B_Δ12_G154C-R396C	A140C, A147C, A249C, V84C, 185P, G154C, R396C	89-112	GSGGSG
	v3B_Δ12_G154gC-R396C	A140C, A147C, A249C, V84C, 185P, G154gC, R396C	89-112	GSGGSG
	v3B_Δ12_G154Cg-R396C	A140C, A147C, A249C, V84C, 185P, G154Cg, R396C	89-112	GSGGSG
	v3B-Δ12_E26-G439	A140C, A147C, A249C, V84C, 185P, E26C, G439C	89-112	GSGGSG
	v3B-Δ12_G70-L375C	A140C, A147C, A249C, V84C, 185P, G70C, L375C	89-112	GSGGSG
	v3B-Δ12_G70gC-L375C	A140C, A147C, A249C, V84C, 185P, G70GC, L375C	89-112	GSGGSG
	v3B-Δ12_T365C-Q455C	A140C, A147C, A249C, V84C, 185P, T365C, Q455C	89-112	GSGGSG
	v3B-Δ12_D454C-V458C	A140C, A147C, A249C, V84C, 185P, D454C, V458C	89-112	GSGGSG
	v3B-Δ12_S293C-S443C	A140C, A147C, A249C, V84C, 185P, S293C, S443C	89-112	GSGGSG
	v3B-Δ12_D66C-R329C	A140C, A147C, A249C, V84C, 185P, D66C, R329C	89-112	GSGGSG
	v3B-Δ12_D66gc-R329	A140C, A147C, A249C, V84C, 185P, D66GC, R329C	89-112	GSGGSG
Cav, charge-charge interaction v3B Δ12	v3B_Δ12_K324E	A140C, A147C, A249C, V84C, 185P, K324E	89-112	GSGGSG
	v3B_Δ12_K324F	A140C, A147C, A249C, V84C, 185P, K324F	89-112	GSGGSG
	v3B_Δ12_K324Q	A140C, A147C, A249C, V84C, 185P, K324Q	89-112	GSGGSG
	v3B_Δ12_V191I	A140C, A147C, A249C, V84C, 185P, V191I	89-112	GSGGSG
	v3B_Δ12_V191I, K324E	A140C, A147C, A249C, V84C, 185P, V191I, K324E	89-112	GSGGSG

Continued on next page.

S1 Table. Engineered RSV F glycoprotein variants, continued.

Rationale	Name	Mutation	F1-F2 Linker residues	Linker sequence
Pro mutation	v3B_Δ12_K143P	A140C, A147C, A249C, V84C, 185P, K143P	89-112	GSGGSG
	v3B_Δ12_E131P	A140C, A147C, A249C, V84C, 185P, E131P	89-112	GSGGSG
	v3B_Δ12_N145P	A140C, A147C, A249C, V84C, 185P, N145P	89-112	GSGGSG
	v3B_Δ12_R163P	A140C, A147C, A249C, V84C, 185P, R163P	89-112	GSGGSG
	v3B_Δ12_ E131P-R163P	A140C, A147C, A249C, V84C, 185P, E131P, R163P	89-112	GSGGSG
	v3B_Δ12_A459P	A140C, A147C, A249C, V84C, 185P, A459P	89-112	GSGGSG
Combination of Pro muts	v3B_Δ12_E131P-K143P-N145P-R163P-A459P	A140C, A147C, A249C, V84C, 185P,E131P, K143P, N145P, R163P, A459P	89-112	GSGGSG
	v3B_Δ12_K143P-N145P-R163P-A459P	A140C, A147C, A249C, V84C, 185P, N145P, R163P, A459P	89-112	GSGGSG
	v3B_Δ12_K143P-N145P-R163P	A140C, A147C, A249C, V84C, 185P, K143P, N145P, R163P	89-112	GSGGSG
	v3B_Δ12_K143P-R163P	A140C, A147C, A249C, V84C, 185P, K143P, R163P	89-112	GSGGSG
	v3B_Δ12_K143P-R163P-E131P	A140C, A147C, A249C, V84C, 185P,E131P, K143P, N145P, R163P, A459P	89-112	GSGGSG
	v3B_Δ12_K143P-R163P-A459P	A140C, A147C, A249C, V84C, 185P, K143P, R163P, A459P	89-112	GSGGSG
	v3B_Δ12_K143P-R163P-N145P	A140C, A147C, A249C, V84C, 185P, K143P, N145P, R163P	89-112	GSGGSG
	v3B_Δ12_E131P-K143P-N145P-R163P	A140C, A147C, A249C, V84C, 185P,E131P, K143P, N145P, R163P	89-112	GSGGSG
	v3B_Δ12_E131P-K143P-R163P-A459P	A140C, A147C, A249C, V84C, 185P,E131P, K143P, R163P, A459P	89-112	GSGGSG
	v3B_Δ12_E131P-N145P-R163P-A459P	A140C, A147C, A249C, V84C, 185P,E131P, N145P, R163P, A459P	89-112	GSGGSG
	v3B_Δ12_E131P-R163P-A459P	A140C, A147C, A249C, V84C, 185P,E131P, R163P, A459P	89-112	GSGGSG
	v3B_Δ12_E131P-R163P-N145P	A140C, A147C, A249C, V84C, 185P,E131P, K143P, N145P, R163P, A459P	89-112	GSGGSG
Combination of Pro muts with DS	v3B_Δ12_R163P-A459P_D454C-V458C	A140C, A147C, A249C, V84C, 185P,R163P, A459P, D454C, V458C	89-112	GSGGSG
	v3B_Δ12_R163P-A459P_T365-Q455	A140C, A147C, A249C, V84C, 185P,R163P, A459P, T365C, Q455C	89-112	GSGGSG
	v3B_Δ12_ E131P-R163P-A459P_D454C-V458C	A140C, A147C, A249C, V84C, 185P,E131P, R163P, A459P,D454C, V458C,T365C, Q455C	89-112	GSGGSG
	v3B_Δ12_ E131P-R163P-A459P_ T365-Q455	A140C, A147C, A249C, V84C, 185P,E131P, R163P, A459P,T365C, Q455C	89-112	GSGGSG
