# Supplementary material for: Structure-based design of a single-chain triple-disulfide-stabilized fusion-glycoprotein trimer that elicits high-titer neutralizing responses against human metapneumovirus
Source: PLoS Pathog. 2023 Sep 22;19(9):e1011584. doi: 10.1371/journal.ppat.1011584 (PMC10516418; doi:10.1371/journal.ppat.1011584)
Supplement: S2 Table — (PDF) [file ppat.1011584.s002.pdf]

S2 Table. Binding affinity of HMPV designs with additional disulfide bonds and proline substitutions.

	MPF5			MPE33			MPE8		
**Sample**	K_D (nM)	k_{on} ($M^{-1}s^{-1}$) x 10^5	k_{off} (s^{-1}) x 10^{-4}	K_D (nM)	k_{on} ($M^{-1}s^{-1}$) x 10^5	k_{off} (s^{-1}) x 10^{-4}	K_D (nM)	k_{on} ($M^{-1}s^{-1}$) x 10^5	k_{off} (s^{-1}) x 10^{-3}
HMPV v3BΔ12_D454C-V458C	0.71±0.01	1.71±0.01	4.22±0.08	3.37±0.01	2.12±0.01	7.14±0.01	8.1±0.1	2.25±0.03	1.82±0.01
HMPV v3BΔ12_T365C-Q455C	0.76±0.07	3.67±0.01	2.78±0.08	2.86±0.01	2.53±0.01	7.25±0.01	25.3± 0.3	1.63±0.02	4.13±0.01
HMPV v3B_D454C-V458C	0.57±0.01	3.70±0.50	2.10± 0.3	9.00±0.30	1.53±0.04	13.8±0.01	No binding		
HMPV v3B_T365C-Q455C	0.60±0.01	3.80±0.50	2.61± 0.3	3.10±0.30	2.5±0.04	8.10±0.01	No binding		
HMPV_v3BΔ12_K143P	1.08±0.02	10.2±0.01	4.22±0.08	1.56±0.04	8.88±0.21	13.9±0.01	5.16±0.08	4.67±0.07	2.41±0.01
HMPV_v3BΔ12_E131P	0.35±0.01	10.1±0.12	3.53±0.05	1.70±0.03	5.55±0.08	9.45±0.07	5.56±0.06	4.65±0.04	2.58±0.01
HMPV_v3BΔ12_N145P	0.50±0.02	7.88±0.02	3.90±0.12	1.48±0.02	5.06±0.05	7.50±0.05	6.85±0.09	3.68±0.05	2.52±0.01
HMPV_v3BΔ12_R163P	0.76±0.01	7.92±0.01	6.01±0.05	1.65±0.03	5.50±0.08	9.05±0.07	5.96±0.06	3.86±0.04	2.30±0.01
HMPV_v3BΔ12_E131P-R163P	0.54±0.01	8.04±0.60	4.33±0.4	1.82±0.03	4.81±0.07	8.73±0.07	6. 92±0.13	3.68±0.05	2.54±0.01
HMPV_v3BΔ12_A459P	0.20±0.01	8.98±0.07	1.83±0.3	1.34±0.02	5.82±0.06	7.82±0.05	7.41±0.12	3.61±0.06	2.67±0.01
HMPV_v3Bv12_E131P-R163P-A459	0.77±0.01	5.76± 0.60	4.48±0.4	2.75±0.03	3.47±0.07	9.55±0.07	6.95±0.10	3.05±0.01	2.12±0.01
