# Supplementary material for: Structure-based design of a single-chain triple-disulfide-stabilized fusion-glycoprotein trimer that elicits high-titer neutralizing responses against human metapneumovirus
Source: PLoS Pathog. 2023 Sep 22;19(9):e1011584. doi: 10.1371/journal.ppat.1011584 (PMC10516418; doi:10.1371/journal.ppat.1011584)
Supplement: S3 Table — (PDF) [file ppat.1011584.s003.pdf]

S3 Table. Cryo-EM data collection, refinement and validation statistics for HMPV F in complex with scFv MPE8.

	HMPV F in complex with scFv MPE8 (EMD-28891) (PDB ID: 8F6X)
**Data collection and processing**	
Magnification	105,000
Voltage (kV)	300
Electron exposure (e^{-}/Å^2)	58.06
Defocus range (μm)	-1.0 to -2.5 μm
Pixel size (Å)	0.83
Symmetry imposed	C3
Final particle images (no.)	192259
Map resolution (Å)	3.25
FSC threshold	0.5
**Refinement**	
Initial model used (PDB code)	7LZE
Model resolution (Å)	3.23
FSC threshold	0.5
Map sharpening B factor (Å^2)	-152.1
Model composition	
Non-hydrogen atoms	14952
Protein residues	1980
Ligands	3 NAG
B factors (Å^2)(mean)	
Protein	49.5
Ligand	79.1
R.m.s. deviations	
Bond lengths (Å)	0.002
Bond angles (°)	0.507
Validation	
MolProbity score	1.86
Clash score	8.9
Poor rotamers (%)	0.06
Ramachandran plot	
Favored (%)	94.29
Allowed	5.66
Disallowed	0.05
