# Supplementary material for: Structure-based design of a single-chain triple-disulfide-stabilized fusion-glycoprotein trimer that elicits high-titer neutralizing responses against human metapneumovirus
Source: PLoS Pathog. 2023 Sep 22;19(9):e1011584. doi: 10.1371/journal.ppat.1011584 (PMC10516418; doi:10.1371/journal.ppat.1011584)
Supplement: S4 Table — (PDF) [file ppat.1011584.s004.pdf]

S4 Table. Serum neutralization of HMPV subtype A2 and B2 strain.

	NHP#	Initial dilution	Serial dilution	$PRNT_{60}$	$PRNT_{60}$ (Log_2)	$PRNT_{60}$	$PRNT_{60}$ (Log_2)
				HMPV A	HMPV A	HMPV B	HMPV B
Group1 prefusion pre bleed	1	1:10	4	63	6	14.2	3.8
	2	1:10	4	15	3.9	<10	<3.3
	3	1:10	4	13	3.7	<10	<3.3
	4	1:10	4	29	4.9	10.4	3.4
	5	1:10	4	23	4.5	10.5	3.4
Group2 postfusion pre bleed	1	1:10	4	78	6.3	<10	<3.3
	2	1:10	4	15	3.9	<10	<3.3
	3	1:10	4	14	3.8	<10	<3.3
	4	1:10	4	<10	<3.3	<10	<3.3
	5	1:10	4	14	3.8	<10	<3.3
Group1 prefusion week 2	1	1:10	4	12648	13.6	1863.8	10.9
	2	1:10	4	3121	11.6	2025.1	11.0
	3	1:10	4	3518	11.8	3420	11.7
	4	1:10	4	9252	13.2	8970.6	13.1
	5	1:10	4	6848	12.7	1831.6	10.8
Group2 postfusion week 2	1	1:10	4	4538	12.1	2415.9	11.2
	2	1:10	4	3925	11.9	1210.6	10.2
	3	1:10	4	5514	12.4	1922.9	10.9
	4	1:10	4	3108	11.6	556.8	9.1
	5	1:10	4	7456	12.9	633.7	9.3
Group1 prefusion week 6	1	1:10	4	17500	14.1	2406.7	11.2
	2	1:10	4	5629	12.5	2083.8	11.0
	3	1:10	4	4804	12.2	2764	11.4
	4	1:10	4	12278	13.6	8861.8	13.1
	5	1:10	4	10993	13.4	2386.4	11.2
Group2 postfusion week 6	1	1:10	4	7920	13	1568	10.6
	2	1:10	4	9544	13.2	1719.8	10.7
	3	1:10	4	9614	13.2	1665.5	10.7
	4	1:10	4	9835	13.3	1631.2	10.7
	5	1:10	4	9040	13.2	2133.5	11.1
Virus only					<3.3		<3.3
Negative control					<3.3		<3.3
Positive control				1143	10.2	88	6.5
