# Supplementary figures and images for: Structure-based design of a single-chain triple-disulfide-stabilized fusion-glycoprotein trimer that elicits high-titer neutralizing responses against human metapneumovirus
Source: PLoS Pathog. 2023 Sep 22;19(9):e1011584. doi: 10.1371/journal.ppat.1011584 (PMC10516418; doi:10.1371/journal.ppat.1011584)

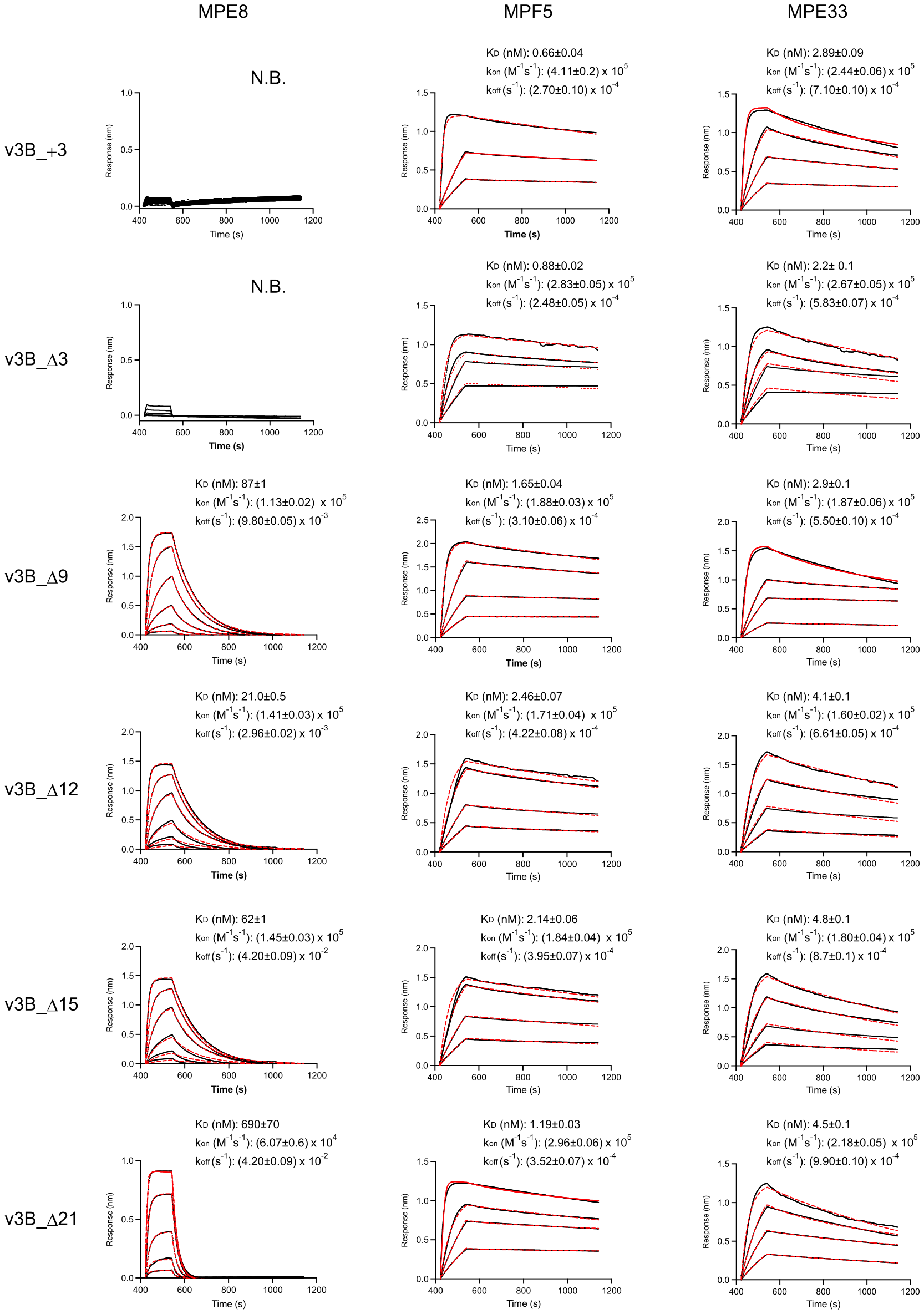

Supplement: S1 Fig — (TIF) [file ppat.1011584.s005.tif]

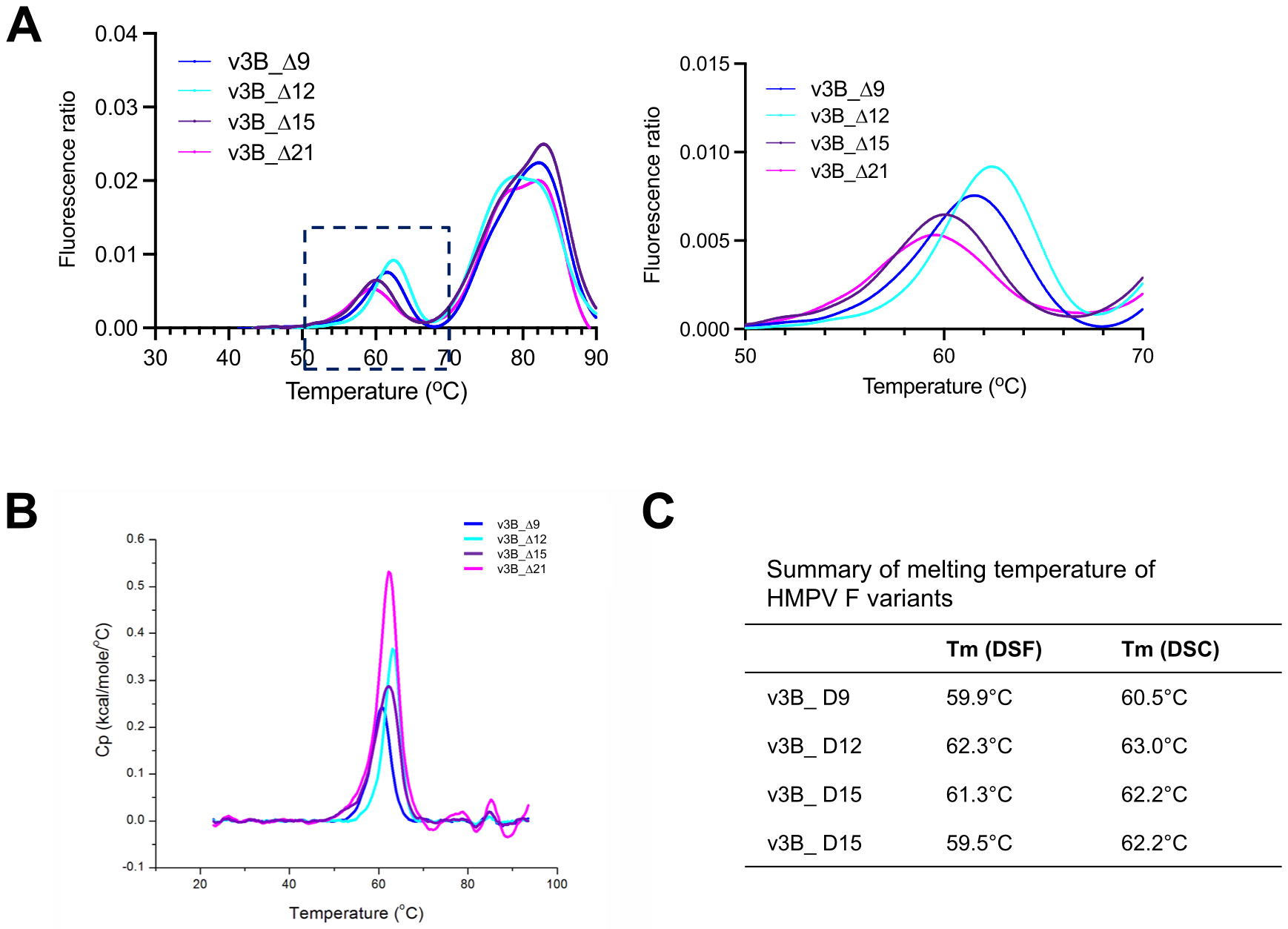

Supplement: S2 Fig — The melting temperature of variants was determined by (A) Nano Differential Scanning Fluorimetry (NanoDSF) and (B) Differential scanning calorimetry (DSC). (C) Summary of melting temperature of HMPV F variants. (TIF) [file ppat.1011584.s006.tif]

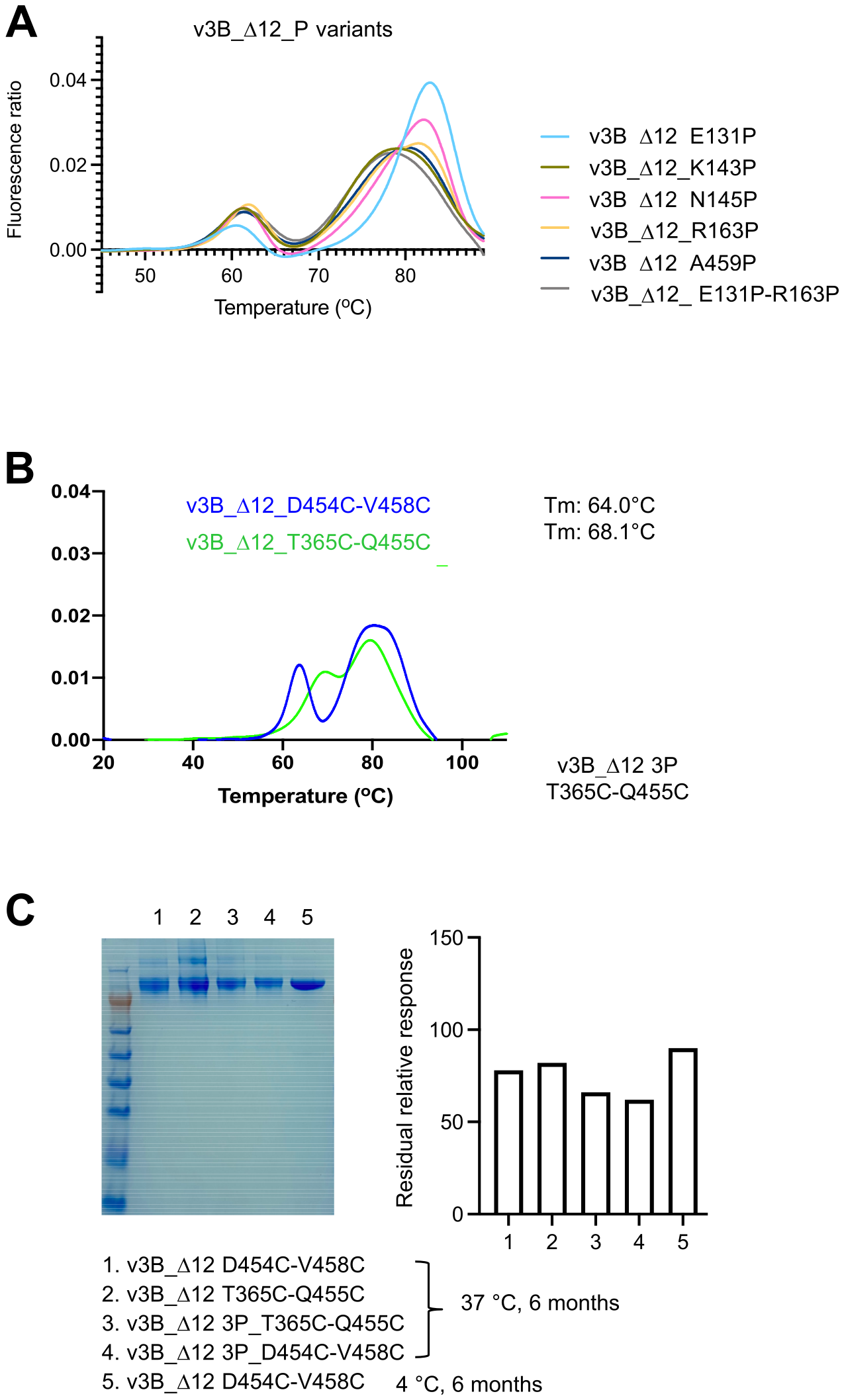

Supplement: S3 Fig — Variants with proline substitution were shown in panel (A) and variants with disulfide bonds were shown in (B). (C) SDS-PAGE and antigenic analysis of disulfide and 3P stabilized HMPV F variants after incubation at 37°C for the indicated time. (TIF) [file ppat.1011584.s007.tif]

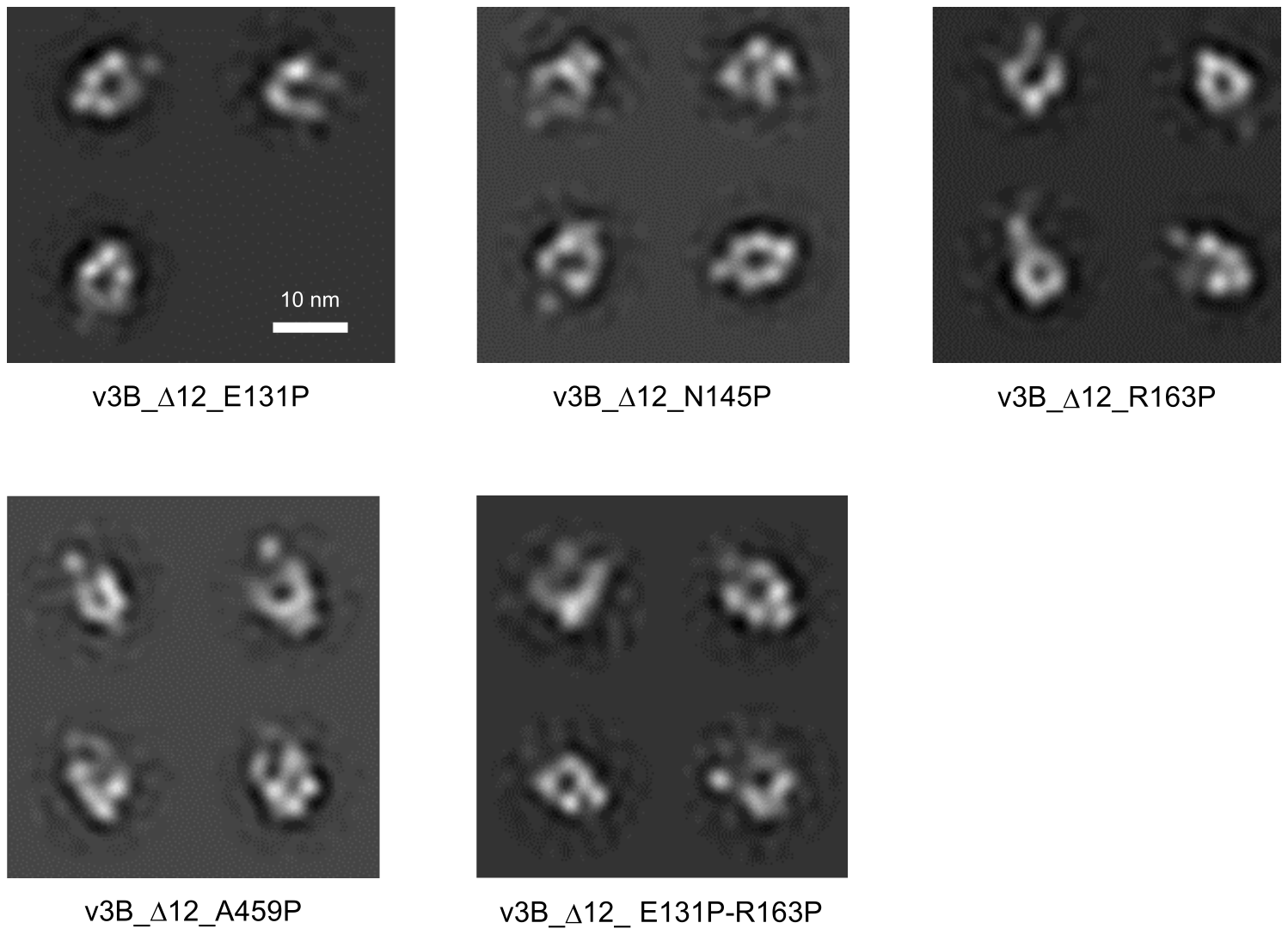

Supplement: S4 Fig — (TIF) [file ppat.1011584.s008.tif]

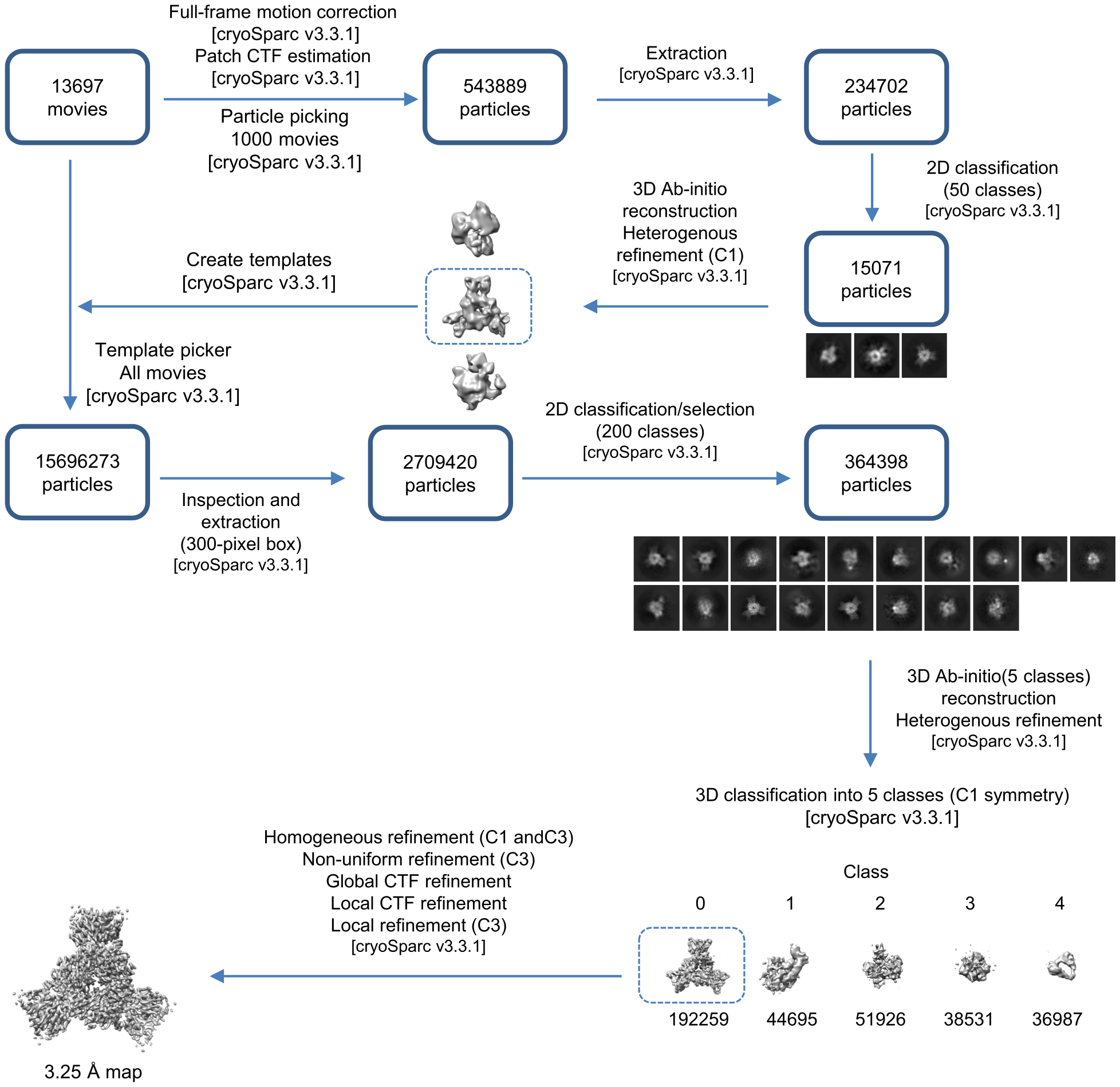

Supplement: S5 Fig — (TIF) [file ppat.1011584.s009.tif]

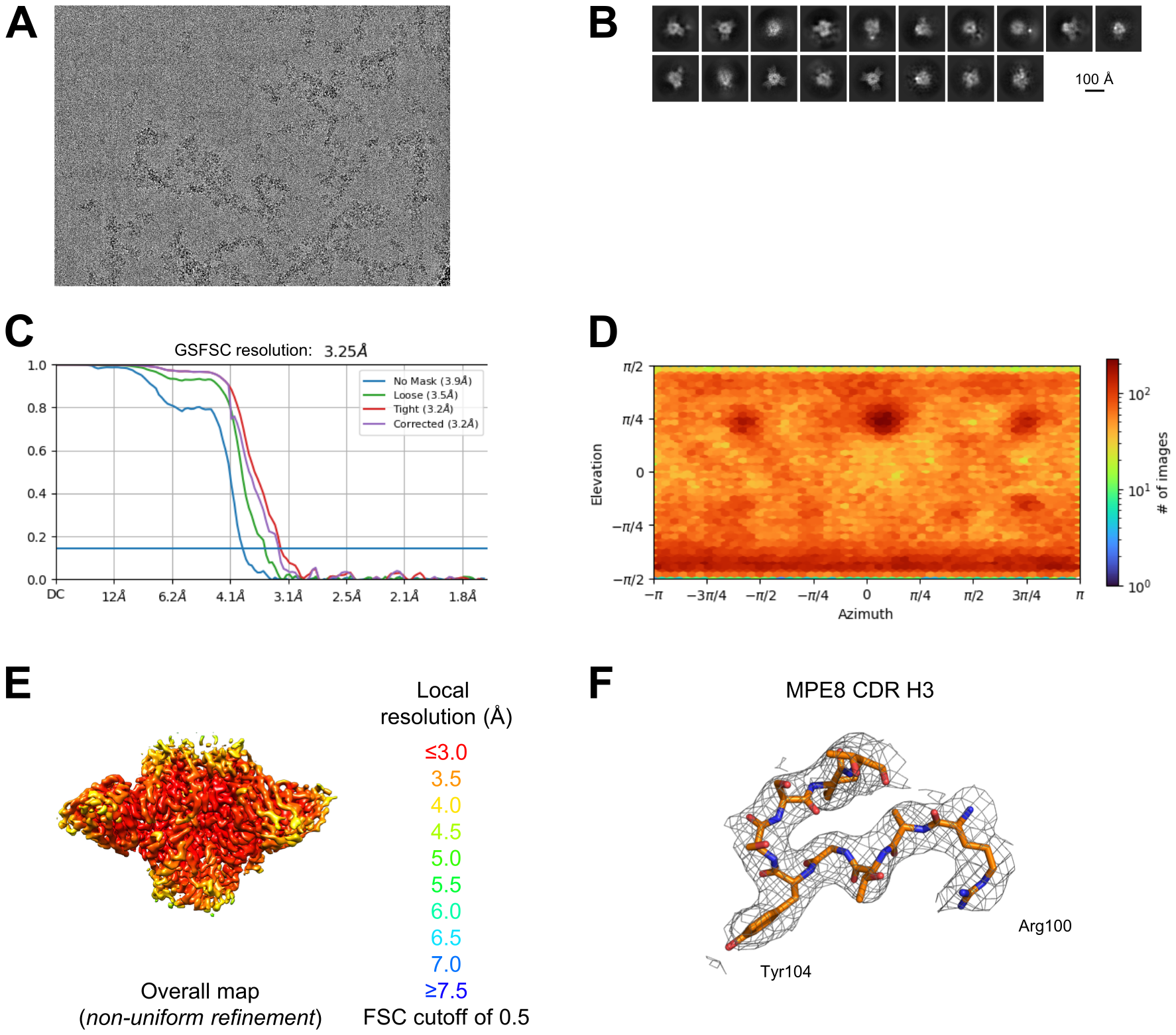

Supplement: S6 Fig — (A) Representative micrograph of HMPV F in complex with scFv MPE8. (B) Representative 2D class averages are shown. (C) The gold-standard Fourier shell correlation resulted in a resolution of 3.25 Å for the overall map using non-uniform refinement with C3 symmetry (left panel); the orientations of all particles used in the final refinement are shown as a heatmap (right panel). (D) The local resolution of the final overall map is shown contoured at 0.448 (5.6s). Resolution estimation was generated through cryoSPARC using an FSC cutoff of 0.5. (E) Representative density is shown for the interface between CDR H3 and HMPV F. The contour level is 1.5σ. (TIF) [file ppat.1011584.s010.tif]

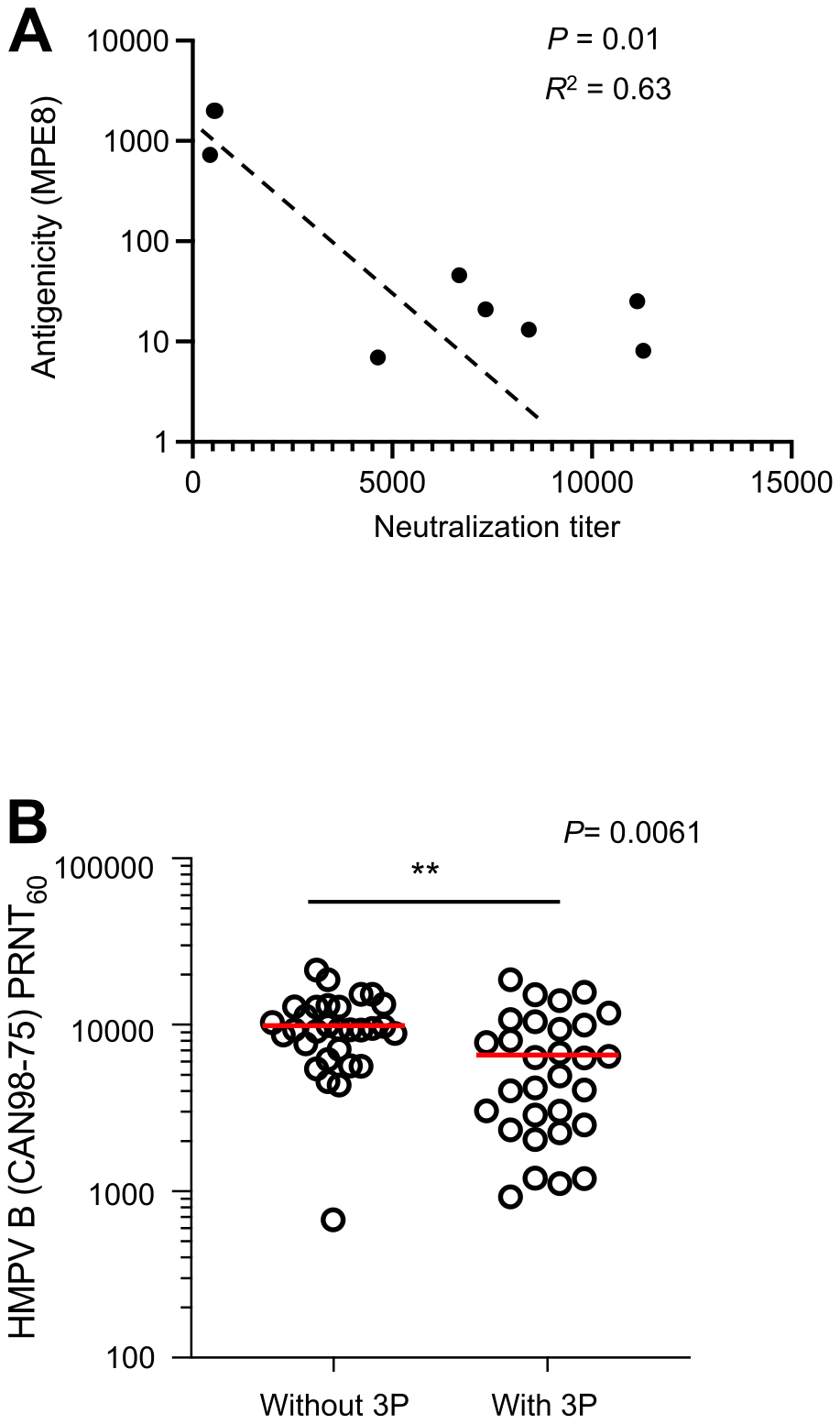

Supplement: S7 Fig — (A) Correlation of antigenicity and immunogenicity of HMPV F variants. (B) Comparison of titers elicited by single chain prefusion stabilized with and without 3P mutations. (TIF) [file ppat.1011584.s011.tif]

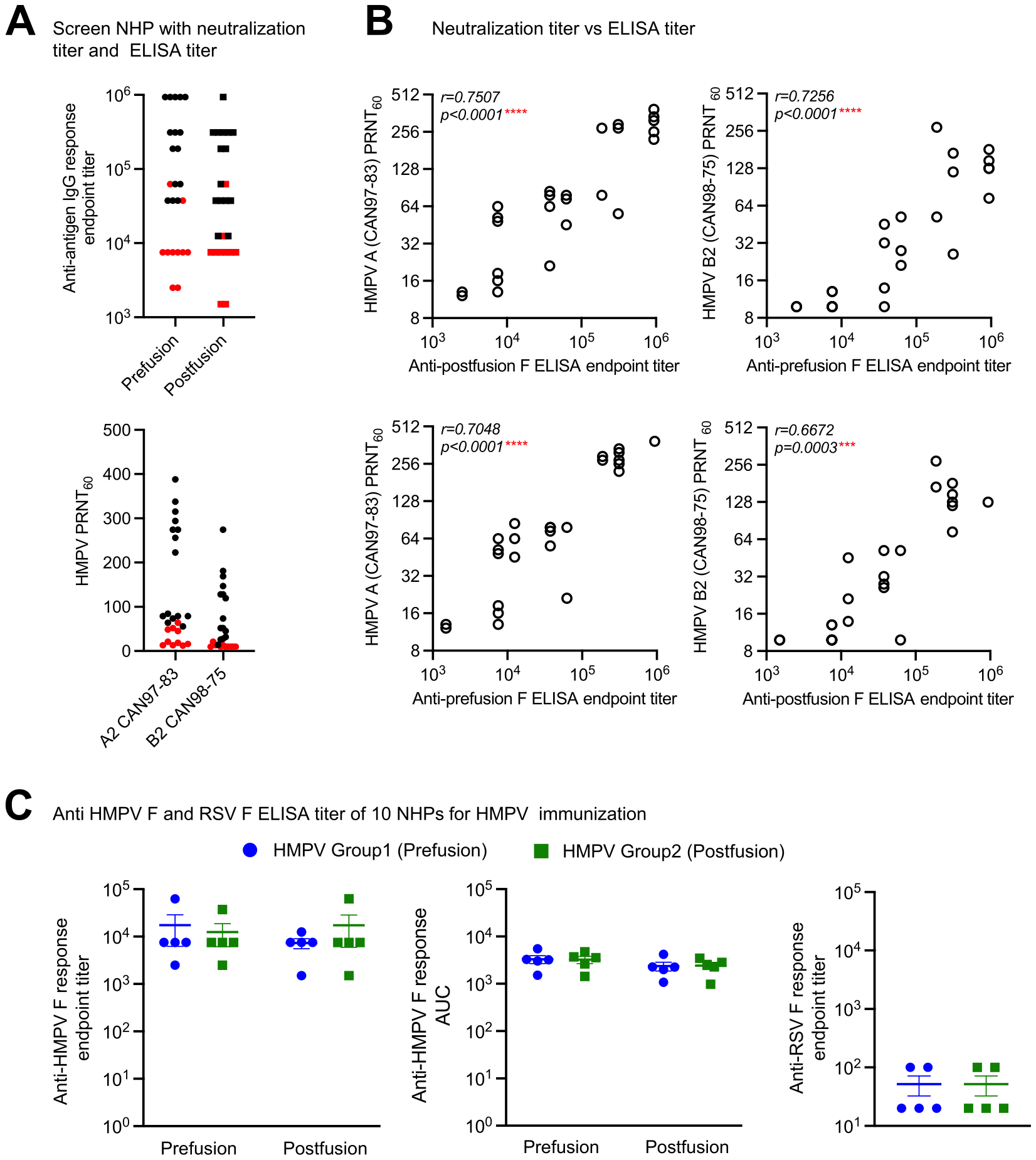

Supplement: S8 Fig — (A) Pre-exposed titers were measured using pre-bleeding by ELISA against HMPV F prefusion (HMPV-F v3B D12_D454C-V458C) and postfusion(postF) immunogens. Neutralization titer were measured by plaque reduction neutralization tests (PRNT) using HMPV subtype A and B strain. 10 NHPs highlighted as color red were selected for HMPV F immunization. (B) Measured anti-F ELISA titer is corelated with neutralization titers. (C) 10 selected NHPs were assigned to prefusion group (blue) and postfusion group (green); ELISA IgG responses with area under curve (AUC) and endpoint titers of each group was shown. Pre-exposed titers against RSV were measured by ELISA with IgG endpoint titers against RSV F DS-Cav1 (right panel). (TIF) [file ppat.1011584.s012.tif]

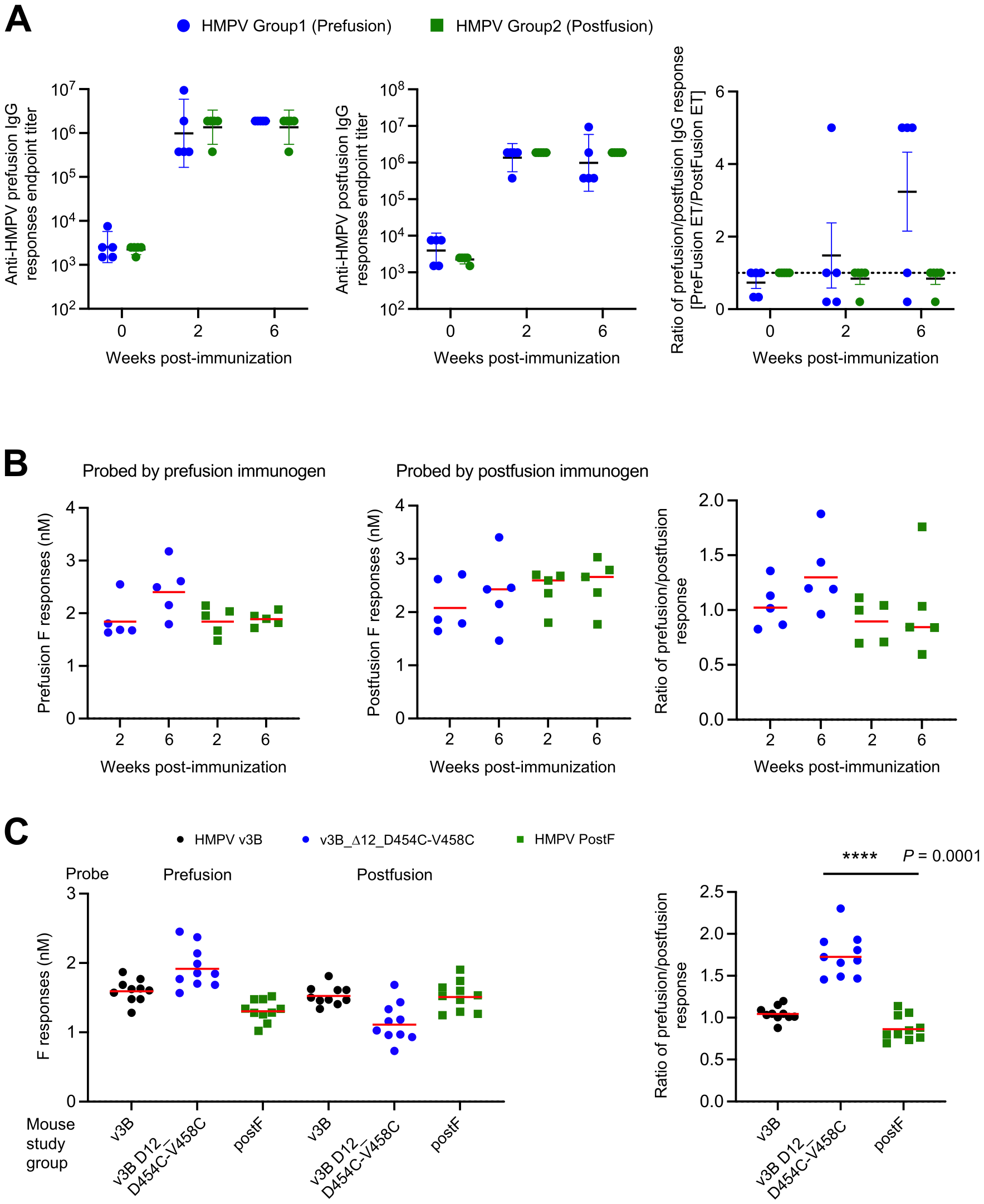

Supplement: S9 Fig — (A) Anti-HMPV prefusion and postfusion IgG responses of NHP sera were measured by ELISA against HMPV F prefusion or postfusion immunogens. Ratios of prefusion vs postfusion were in right panel. (B) Anti-HMPV prefusion and postfusion responses of NHP sera were probed by Octet. (C) Three mouse study groups were selected, and anti-HMPV prefusion and postfusion responses were probed by Octet. (TIF) [file ppat.1011584.s013.tif]
